# Intranodal expansion of follicular T helper cells in patients with multiple sclerosis

**DOI:** 10.1172/jci.insight.188125

**Published:** 2025-10-08

**Authors:** Joona Sarkkinen, Eliisa Kekäläinen, Leo Hannolainen, Ada Junquera, Johannes Dunkel, Maria F. Perdomo, Mikko I. Mäyränpää, Sini M. Laakso

**Affiliations:** 1 Translational Immunology Research Program and; 2 Department of Bacteriology and Immunology, University of Helsinki, Helsinki, Finland.; 3 HUS Diagnostic Center, Clinical Microbiology, Helsinki University Hospital, Helsinki, Finland.; 4 Department of Virology, University of Helsinki and Helsinki University Hospital, Helsinki, Finland.; 5 Research Program in Systems Oncology and; 6 Department of Pathology, University of Helsinki, Helsinki, Finland.; 7 Pathology, HUS Diagnostic Center, Helsinki, Finland.; 8 Department of Neurology, Brain Center, Helsinki University Hospital, Helsinki, Finland.

**Keywords:** Autoimmunity, Immunology, Autoimmune diseases, Multiple sclerosis

## Abstract

The efficacy of anti-CD20 therapies places B cells and their interaction with T cells at the center of attention for multiple sclerosis (MS) pathogenesis. Follicular T helper cells (Tfh), which guide B cell maturation in germinal centers within lymph nodes (LNs), are elevated in the circulation and cerebrospinal fluid of patients with MS (pwMS). However, the LN spatial landscape has remained largely without investigation for pwMS. Using cyclic immunofluorescence, we assessed cell abundance and spatial connections in FFPE LNs of 33 pwMS and 35 non-MS controls. The presence of EBV was analyzed through EBER immunostaining and multiplex quantitative PCR. Our analysis showed that Tfh cells were expanded in LNs of pwMS and accumulated especially in the mantle zone and B cell follicles compared with controls. The Tfh/T follicular regulator ratio was increased in pwMS, while B cell ratios were similar between the cohorts. The interaction of Tfh cells with follicular B cells was higher in pwMS. Interestingly, Tfh accumulation was also observed in 5 prediagnostic MS cases. No signs of EBV latency were detected in either group. These findings highlight LNs as a site of early and persistent immune activation in pwMS, with therapeutic implications to be further addressed.

## Abstract 2

Follicular T helper cells are increased in lymph nodes of patients with multiple sclerosis.

## Introduction

Multiple sclerosis (MS) is a T cell–mediated autoimmune disease of the central nervous system (CNS), but the high efficacy of B cell–depleting therapies (1) has placed the interaction of T and B cells at the forefront of disease pathology. Concurrently, EBV infection has been shown to be a prerequisite for MS (2). Since EBV infection and latency depend upon residence within B cells taking place in secondary lymphoid organs (SLOs), such as lymph nodes (LNs) (3), these tissues present a potentially critical microenvironment where early immunopathological events relevant to MS could be initiated.

Previous studies focusing on LNs in MS are limited. Expanded B cell clones within the inflammatory lesions of the CNS of patients with MS (pwMS) originate from the deep cervical lymph nodes (dcLNs) (4), and dcLNs are reported to be enlarged in these individuals (5). The glymphatic system of the brain drains CNS-derived antigens to the dcLNs (6, 7), where myelin protein has been detected in pwMS (8, 9). In other autoimmune neurological diseases, autoantigen-specific memory B cells are enriched within dcLNs (10, 11), suggesting these nodes may act as hubs for CNS-directed immune responses.

Recently, our group demonstrated significant changes in multiple T and B cell subpopulations in dcLNs of pwMS, pointing to altered germinal center (GC) reactions (12). GCs are specialized structures where T-B cell interactions shape the antigen specificity and function of memory B cells and plasma cells. Within GCs, B cells undergo somatic hypermutation and affinity maturation, processes dependent on help from follicular T helper (Tfh) cells (13, 14). In pwMS, circulating Tfh cells are increased and correlate with disease activity (15–17). Additionally, the ratio of Tfh to T follicular regulator (Tfr) cells is elevated in the blood of pwMS and is associated with increased intrathecal IgG production (18). The diagnostic hallmark of MS is the intrathecal detection of a wide range of antibodies produced by somatically hypermutated B cells (19), further pointing toward a role for the GC reaction. Chronic inflammation in LNs, such as persistent viral infection, can disrupt Tfh–GC B cell interactions and reduce Tfr cell–mediated control over autoreactive B cells (20, 21).

Although T-B cell interactions are known to play a central role in MS pathogenesis, little is known about the spatial organization and frequency of follicular T and B cell subsets within the LN microenvironment in pwMS. Therefore, we analyzed FFPE LNs from pwMS and non-MS controls from the Helsinki Biobank using cyclic multiplex immunofluorescence (mfIHC) and single-cell imaging. We observed an increased abundance of Tfh cells in pwMS compared with controls, and this imbalance was already detectable in samples collected before clinical MS diagnosis.

## Results

### Characteristics of the dataset and main cell subtypes.

**Figure 1:**
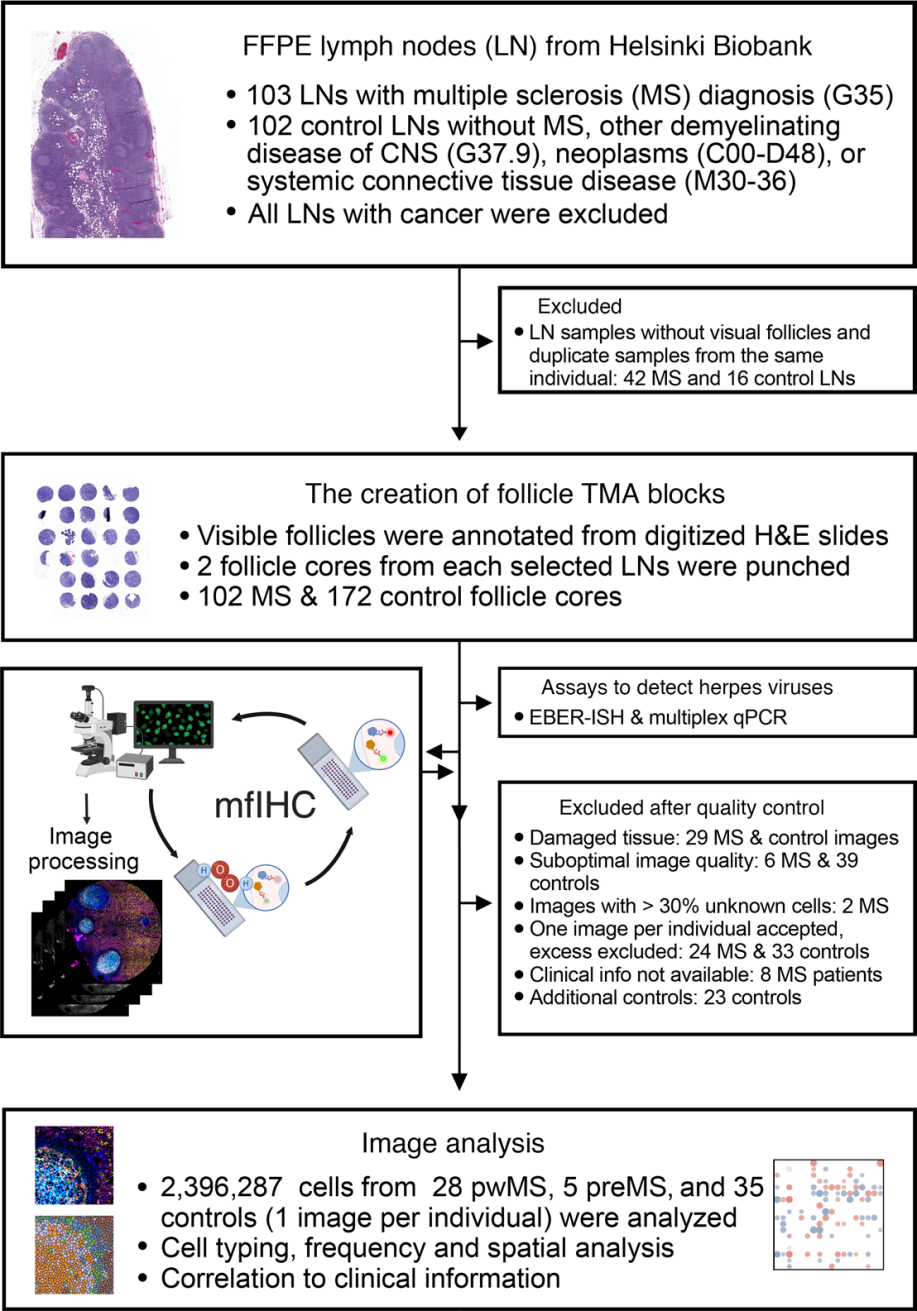
The study design. A flow diagram shows the sample selection and illustrates the analysis workflow. H&E, hematoxylin and eosin; pwMS, patients with MS, sample taken after MS diagnosis; preMS, sample taken before clinical MS diagnosis; qPCR, quantitative PCR.

Archival LN samples from 61 pwMS and 86 controls were collected from the Helsinki Biobank (Figure 1 and Supplemental Figure 1A; supplemental material available online with this article; https://doi.org/10.1172/jci.insight.188125DS1). For downstream analyses, B cell–rich follicles from each sample were selected to construct tissue microarrays (TMAs), enabling high-throughput comparison across the cohort. After exclusion of, e.g., low-quality images (Figure 1), we used data from 28 pwMS who were diagnosed with MS after LN removal (mean disease duration 15 years), 5 individuals from whom the LN was taken before MS diagnosis (preMS; mean 5.5 years preceding), and 35 non-MS controls. Most of the pwMS had either relapsing-remitting MS (RRMS) (52%) or secondary progressive MS (SPMS) (39%), while 3 patients (9%) had primary progressive MS (PPMS). The majority of LNs were axillary (82% in pwMS and 89% in controls) and originally removed for diagnostic purposes, most commonly because of local malignancy of the breast. Controls were matched for age, sex, anatomical location, and presence of malignancy (Table 1 and Supplemental Table 1).

To investigate whether MS-related immune dysregulation could be detected in the LN microenvironment, we performed mfIHC on B cell follicles from 33 pwMS and 35 matched controls (Figure 1). After artifact removal, over 2.3 million single cells were identified and analyzed (Supplemental Figure 1, B–E). The major immune cell types included CD4^+^ T helper (Th) cells, FOXP3^+^ regulatory T (Treg) cells, CD8^+^ T (Tc) cells, CD20^+^ B cells, follicular dendritic cells, epithelial cells, and Pnad^+^ high endothelial venules. No significant differences were observed in the abundance of these major cell types between pwMS and controls, clinical variables, or anatomical location of the LNs, when normalized to tissue area (Supplemental Figure 1, F and G).

### Tfh cells are increased in LNs of pwMS.

**Figure 2:**
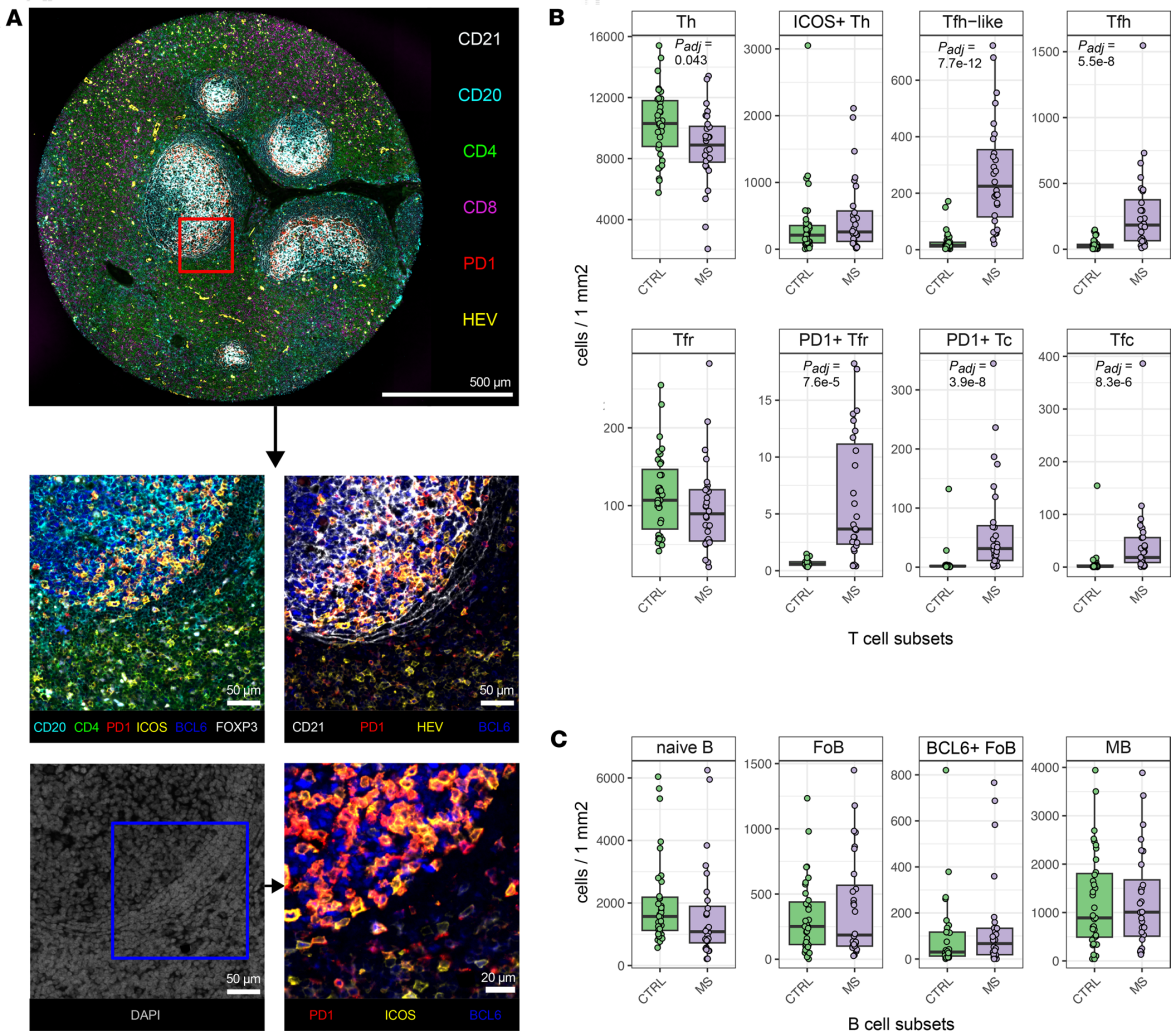
Tfh cells are increased in pwMS. (**A**) mfIHC images with scale bars demonstrate the localization of the cell types of interest with the selected markers. Images in the middle and bottom left are magnified from the top image (red rectangle), whereas the bottom right image is magnified from the bottom left (blue rectangle). The orange color in the bottom right image shows PD-1^+^ (red) and ICOS^+^ (yellow) Tfh cells with nuclear BCL6^+^ (blue) staining. (**B**) Box plots of Th, Treg, and Tc cell subsets between pwMS and controls. (**C**) Box plots of B cell subsets between pwMS and controls. Box plots show the interquartile range, median (line), and minimum and maximum (whiskers). The Benjamini-Hochberg–corrected (BH-corrected) unpaired Wilcoxon test was used in **B** and **C**, and *P_adj_* values < 0.05 are shown.

We next focused on more specific subsets of T and B cells, analyzing LNs from the 35 controls and the 28 pwMS whose LNs were collected after MS diagnosis. Follicular T cells were defined based on expression of programmed cell death 1 (PD-1), ICOS, and BCL6, which are characteristic markers of Tfh and Tfr cells (13), though Tfr cells may also be PD-1 negative (22) (Figure 2A). Tfh cells were defined as PD-1^+^BCL6^+^ICOS^+/–^ Th cells. We also identified a population of follicular PD-1^+^BCL6^–^ Th cells that we named “Tfh-like” cells. These cells potentially represent early (pre-Tfh) or noncanonical Tfh cells that have not yet fully upregulated BCL6 (23, 24). Although CXCR5 is a canonical marker for follicular localization, its inconsistent staining in archival samples limited its use; nonetheless, the spatial analysis verified follicular positioning of these cells. The pwMS had more Tfh (*P_adj_* = 5.5 × 10^–8^) and Tfh-like cells (*P_adj_* = 7.7 × 10^–12^) per square millimeter than controls, alongside a modest reduction in PD-1^–^BCL6^–^ICOS^–^ Th cells (*P_adj_* = 0.043) (Figure 2B). Numbers of ICOS^+^ Th cells, potentially representing activated nonfollicular Th cells, were unchanged.

Among Treg cells, PD-1^+^BCL6^+^ cells (considered Tfr cells, *P_adj_* = 7.6 × 10^–5^) and PD-1^+^BCL6^–^ cells (PD-1^+^ Treg cells, *P_adj_* = 0.0078) were elevated in pwMS, whereas PD-1^–^ Tfr and PD-1^–^BCL6^–^ Treg cells showed no group differences (Figure 2B and Supplemental Figure 2A). Similarly, we identified PD-1^+^BCL6^+^ Tc cells, consistent with follicular cytotoxic T (Tfc) cells (25). These cells were significantly increased in pwMS (*P_adj_* = 4.2 × 10^–6^), as were BCL6^–^PD-1^+^ Tc cells (*P_adj_* = 4.3 × 10^–8^), whereas PD-1^–^ Tc cells showed no significant difference (Figure 2B and Supplemental Figure 2A).

We next analyzed the follicular B cell subsets. CD20^+^ B cells were categorized into BCL6^+^ or BCL6^–^CD21^+^ follicular B (FoB), CD27^+^ memory B (MB), and CD21^–^CD27^–^ naive B cells. However, no significant differences were detected between pwMS and controls (Figure 2C).

LNs contain follicles in 2 main forms: primary follicles, which are composed of resting, naive B cells and lack signs of antigenic stimulation, and secondary follicles, which emerge following antigen exposure and contain active GCs where B cells proliferate, mutate, and undergo selection. Since our dataset had heterogeneity and contained both types, we next classified the images into primary (without GCs) and secondary follicles (with identifiable GCs), based on BCL6, Ki67, and PD-1 expression (Methods and Supplemental Figure 2B). When we analyzed the resting primary follicles, only Tfh-like cells were modestly increased in pwMS, whereas all other analyzed cell populations were equal between pwMS and controls. In secondary follicles, the pwMS had increased numbers of Tfh-like, Tfh, PD-1^+^ Tfr, and PD-1^+^ Tc populations compared with controls (Supplemental Figure 2C).

In summary, pwMS exhibited a clear increase in follicular T cell subsets, particularly Tfh, Tfr, and Tfc-like cells in active GCs, suggesting chronic local immune activation within the LN follicles despite comparable B cell subset distributions. These observations were not dependent on the participants’ possible malignancy, MS type, immunosuppressive treatment, or age (Supplemental Figure 2D).

### Tfh cells accumulate in the mantle zone in pwMS.

**Figure 3:**
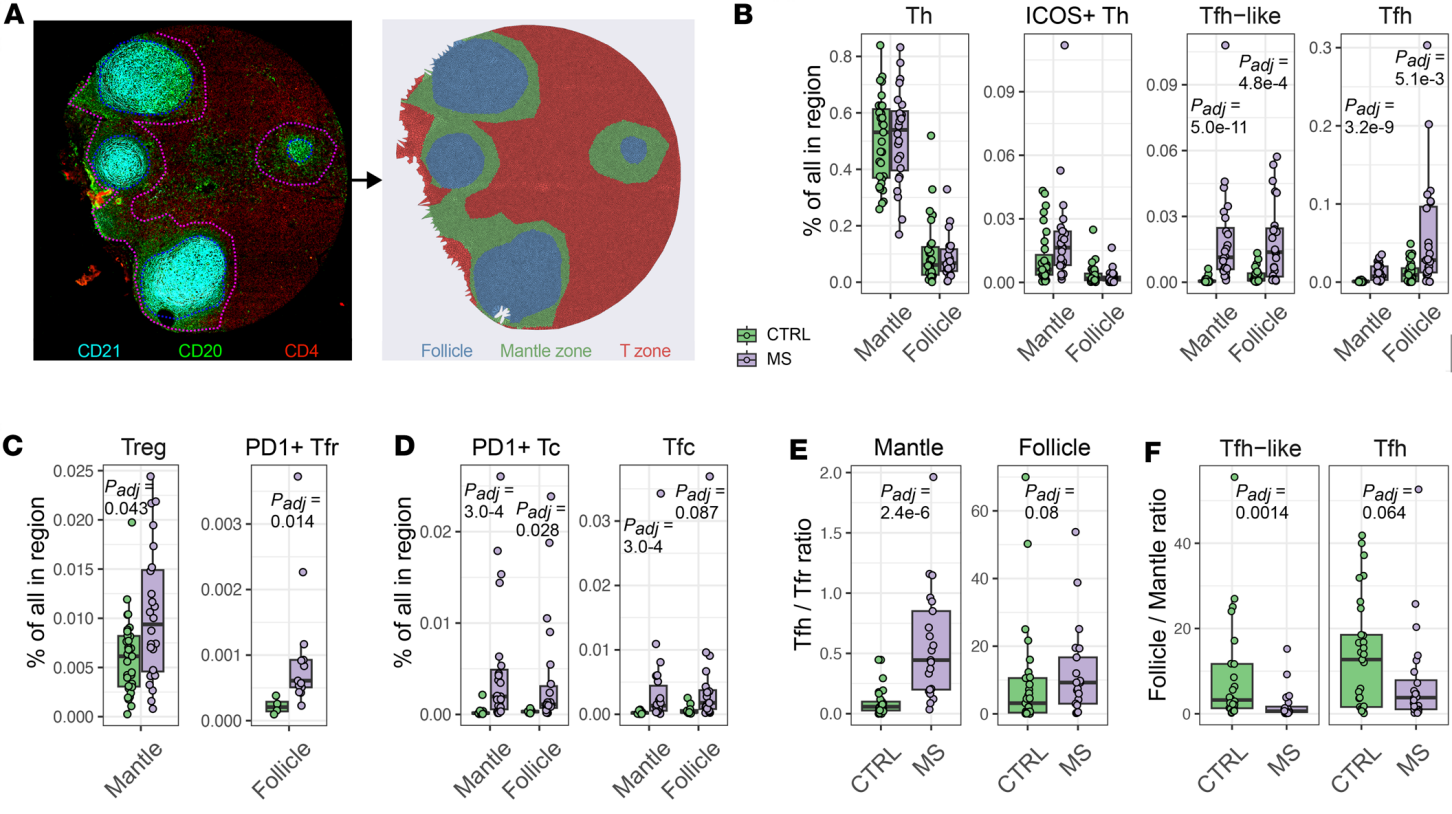
Expanded Tfh cells also accumulate in the mantle zone in pwMS. (**A**) A multiplex immunofluorescent image and Voronoi plot demonstrate the spatial analysis. (**B**–**D**) Frequencies of (**B**) CD4^+^ T cell, (**C**) FOXP3^+^ T regulatory cell, and (**D**) CD8^+^ T cell subsets in mantle zone and follicle with box plots. (**E**) Box plots of Tfh-to-Tfr ratio in mantle zone and follicle. (**F**) The follicle–mantle zone ratios of Tfh-like and Tfh cells using box plots. Box plots of B cell subsets between pwMS and controls. Box plots show the interquartile range, median (line), and minimum and maximum (whiskers). The BH-corrected unpaired Wilcoxon test was used in **B**–**E**. *P_adj_* < 0.10 are shown.

Tfh cells at different developmental stages localize to distinct regions within the LN follicle. Immature pre-Tfh cells migrate from the T cell–rich zone to the follicular mantle, guided by the stimulation from their cognate B cells. In contrast, fully differentiated effector Tfh cells are predominantly found within the B cell–rich GCs, where they provide contact-dependent help to B cells to generate long-lived humoral responses (23). To dissect if the altered proportions of follicular T cell populations could reflect different developmental trajectories in pwMS, we next analyzed the follicular cell type abundance and spatial proximities in the follicle and mantle zone separately. Since our images included B cell–rich follicles (CD20^+^CD21^+^), surrounding mantle zones (CD20^+^CD21^–^), and adjacent T cell–dominated regions (CD3^+^CD4^+^/CD8^+^), we first manually annotated these areas based on CD21, CD20, and CD4 staining patterns (Figure 3A) and focused our analysis on mantle zones and follicles. To minimize the potential effects of anatomical variation on spatial immune cell composition, we limited the analysis to axillary LNs (26 pwMS and 31 controls).

In both the mantle zone and the follicle, the proportion of Tfh cells (mantle *P_adj_* = 2.5 × 10^–9^, follicle *P_adj_* = 0.0045) and Tfh-like cells (mantle *P_adj_* = 3.8 × 10^–11^, follicle *P_adj_* = 0.00041) was larger in pwMS than in controls, whereas ICOS^+^ Th and PD-1^–^BCL6^–^ICOS^–^ Th cells were present in similar proportions (Figure 3B). Similarly, pwMS had an increased proportion of PD-1^+^ Tfr cells in the follicle (*P_adj_* = 0.013) and PD-1^–^BCL6^–^ Treg cells in the mantle zone (*P_adj_* = 0.043), while PD-1^–^ Tfr cells were comparable between the pwMS and controls (Figure 3C and Supplemental Figure 3A). In addition, Tfc cells and PD-1^+^ Tc cells were more frequent in pwMS in both the mantle zone (*P_adj_* = 0.0003; *P_adj_* = 0.0003) and follicle (*P_adj_* = 0.087; *P_adj_* = 0.0003, Figure 3D). We also observed a reduced proportion of naive B cells in the follicle in pwMS (*P_adj_* = 0.04), while the proportions of other B cell subsets were relatively similar between the cohorts (Supplemental Figure 3B).

As a proxy for Tfr cell control over Tfh cells, we calculated the Tfh-to-Tfr ratio separately within the mantle zones and follicles. For this, all FOXP3^+^ Treg cells were pooled within each respective compartment. The Tfh-to-Tfr ratio was increased in pwMS within the mantle zone (*P_adj_* = 2.4 × 10^–6^), while no difference in this ratio was observed in the follicle between pwMS and controls (Figure 3E). We then compared the relative positioning of follicular T cells between the follicle and mantle zone (follicle-to-mantle ratio). In both cohorts, Tfh and Tfh-like cells were enriched in the follicle; however, pwMS had a lower follicle-to-mantle ratio for Tfh-like cells (*P_adj_* = 0.014) and a trend toward a decreased ratio for Tfh cells (*P_adj_* = 0.064, Figure 3F). In contrast, FOXP3^+^ Treg cells are more frequently located in the mantle zone in both pwMS and control LNs, with no difference between the cohorts (Supplemental Figure 3C). For Tfr subsets specifically, the follicle-to-mantle ratio could not be reliably calculated because of low cell numbers.

Together, these findings suggest altered spatial organization of follicular T cell subsets in pwMS, characterized by accumulation of Tfh-like cells at the mantle zone, most likely reflecting an accumulation of pre-Tfh cells. In the same microanatomical niche, the ratio of Treg cells to follicular effectors was lower in pwMS.

### Tfh cells have increased interactions with FoB cells in MS LNs.

**Figure 4:**
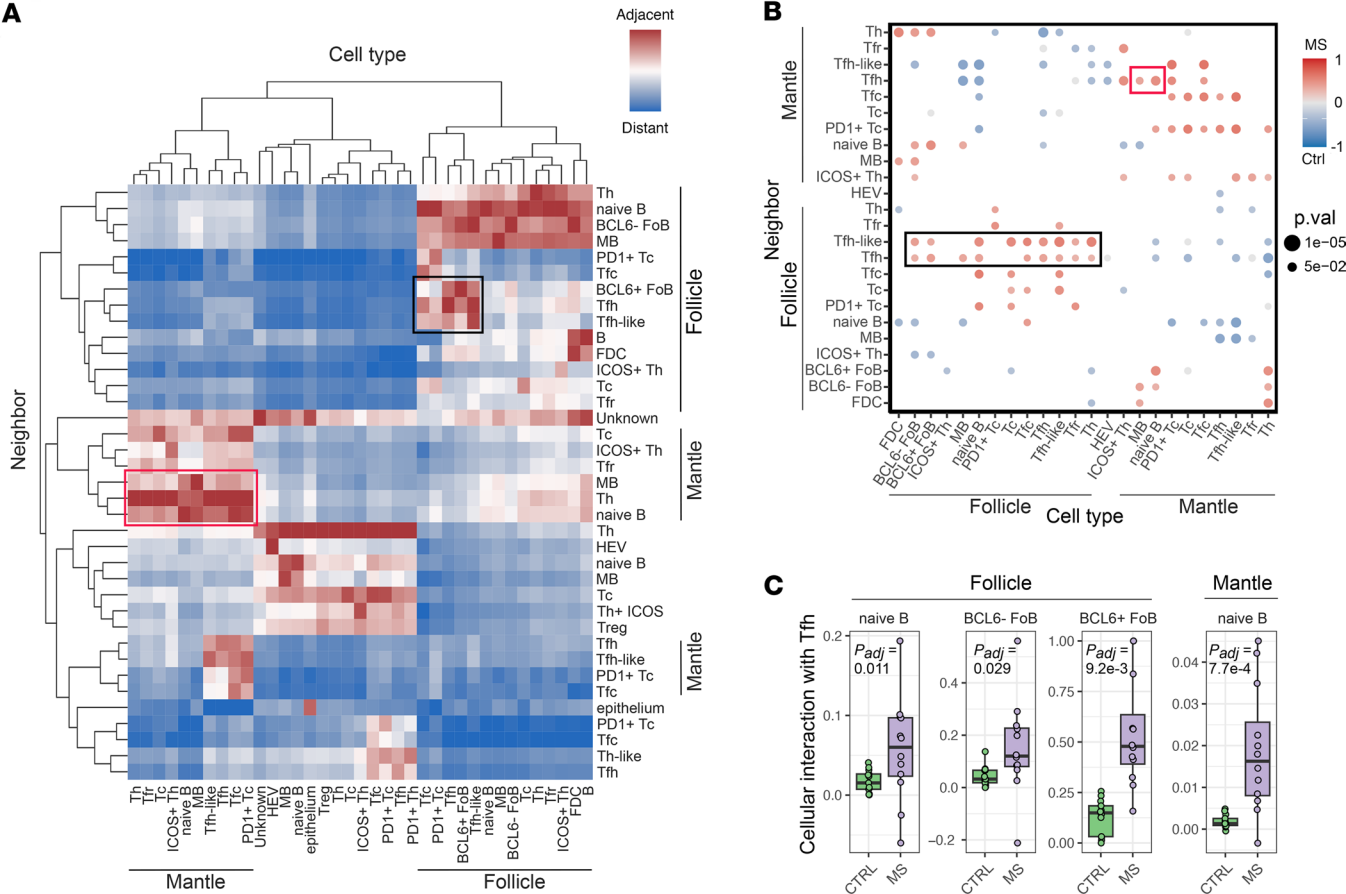
Tfh cells interact more with FoB cells in pwMS. (**A**) Clustered heatmap of average shortest distances between cell types. The cell types were determined using a marker profile and spatial location combination. The black rectangle emphasizes the proximity of FoB, Tfh, and Tfh-like cells in the follicle. The red rectangle highlights that naive B and MB cells are close to several follicular T cell subsets in the mantle zone. (**B**) Spatial interactions between different cell types that are significantly (Wilcoxon *P* value < 0.05) enriched (red) or decreased (blue) in pwMS compared with controls. Insignificant interactions are not shown. Increased spatial interactions in pwMS within follicular T cells and between B and follicular T cells are highlighted with red (follicle) and black rectangles (mantle zone), respectively. (**C**) Spatial interactions between Tfh cells and B cell subsets in the follicle and mantle zone using box plots. Box plots of B cell subsets between pwMS and controls. Box plots show the interquartile range, median (line), and minimum and maximum (whiskers). The BH-corrected unpaired Wilcoxon test was used in **C**.

Given the observed abundance of Tfh and Tfh-like cells in MS LNs, we next analyzed spatial interactions between follicular T cells and B cells, which are crucial for the GC response and its control. First, we calculated the average shortest spatial distances between the cell types, as described previously (26), to comprehend cellular organization in the LN cortex. Each cell’s identity was defined by a combination of marker expression (Figure 2A) and anatomical location (Figure 3A). Notably, within the follicle, Tfh cells, Tfh-like cells, and BCL6^+^ FoB cells were often found in close proximity to each other (Figure 4A). Given that preselection for the GC occurs outside the GC, we observed several follicular T cell subsets near naive B cells and MB cells in the mantle zone. Tfh cells also play a role in activating other T cell subsets (13). Consistent with this, we identified a separate cluster comprising Tfh, Tfh-like, Tfc, and PD-1^+^ Tc cells that were spatially adjacent, suggesting broader roles in shaping T cell responses in LNs.

To evaluate whether the spatial interactions were altered in pwMS, we quantified the 10 spatially nearest neighbors for each cell. We applied a permutation-based test (27) to determine whether certain cell types were more frequently found near others than expected by chance. Tfh and Tfh-like cells exhibited increased spatial interactions between FoB (BCL6^+/–^), naive B, and MB cells, as well as with several follicular T cell subsets in the follicle of pwMS compared with controls. Similarly, Tfh cells had increased interactions with naive and MB cells in the mantle zone in pwMS (Figure 4B).

To focus on follicles with actively ongoing GC reactions — where interactions between Tfh and B cells are biologically most relevant — we restricted the analysis to samples containing GCs with a Ki67-positive staining pattern (i.e., secondary follicles; Supplemental Figure 2C). Here, the FoB cells are considered functionally engaged GC B cells. Within these samples, Tfh cells in pwMS showed stronger spatial interactions with naive B cells both in the mantle zone (*P_adj_* = 0.00065) and in the follicle (*P_adj_* = 0.026) and with BCL6^+^ GC B cells (*P_adj_* = 0.00042) and BCL6^–^ GC B cells (*P_adj_* = 0.0091, Figure 4C). Tfh-like cells had increased interactions with BCL6^+^ GC B cells (*P_adj_* = 0.00051) and BCL6^–^ GC B cells (*P_adj_* = 0.002) in pwMS, suggesting that they may contribute to GC response despite lacking canonical BCL6 expression (Supplemental Figure 4).

### Expansion of Tfh cells is also seen before MS diagnosis.

To explore whether Tfh cell expansion precedes clinical MS onset, we analyzed LNs from 5 individuals who were later diagnosed with MS (preMS). These individuals were 20- to 50-year-old women who either were cancer free or had a local cancer. Later, they were diagnosed with RRMS (*n* = 3), SPMS (*n* = 1), or PPMS (*n* = 1). LNs represented various anatomical locations, and in 3 of the individuals, the LNs were taken within 2 years of MS onset (Supplemental Table 1).

The preMS samples had increased numbers of Tfh-like cells (*P_adj_* = 3.6 × 10^–4^), while Tfh cells showed a trend of elevation compared with controls (*P_adj_* = 0.071). In addition, Tfc (*P_adj_* = 0.022), PD-1^+^ Tc (*P_adj_* = 0.022), PD-1^+^ Treg (*P_adj_* = 0.023), and PD-1^+^ Tfr cells (*P_adj_* = 0.029) were more prevalent in preMS than in controls but present in similar numbers compared to pwMS (Supplemental Figure 5A). In contrast, naive B cells (*P_adj_* = 0.022) were decreased in preMS compared with controls (Supplemental Figure 5B).

Given the heterogeneity of preMS samples, which included images with (*n* = 3) and without (*n* = 2) GCs, we then limited the analysis to only follicles with GCs. In this subset, Tfh-like cells (*P_adj_* = 0.021) and Tfh cells (*P_adj_* = 0.021) remained elevated compared with controls, while other cell types did not differ significantly (Supplemental Figure 5C).

**Figure 5:**
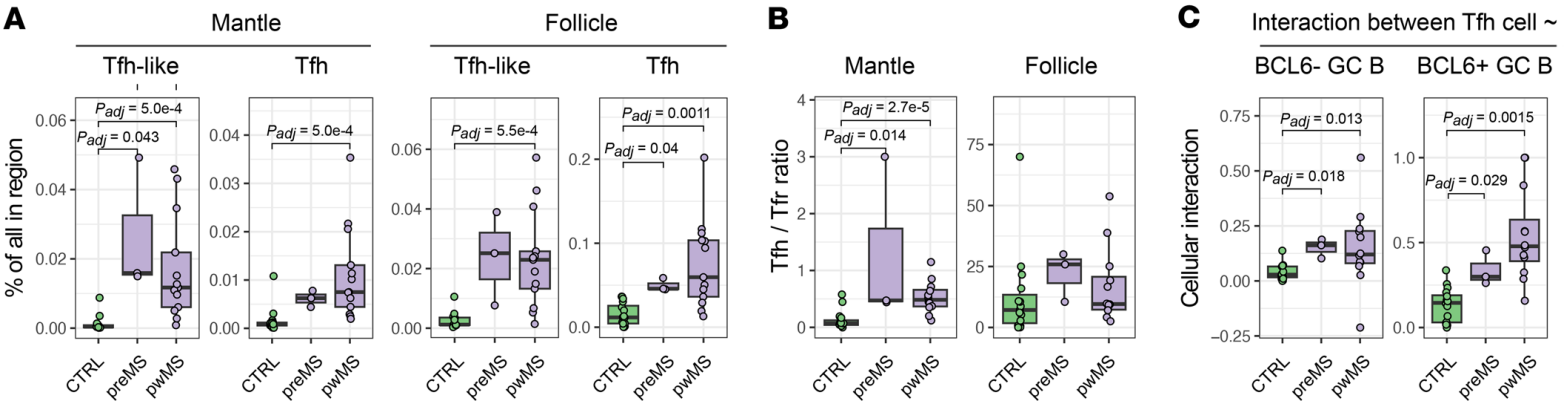
Increased Tfh cells are also in LNs before MS diagnosis. (**A**) Box plots of the proportion of Tfh-like cells and Tfh cells in the mantle zone and follicle in controls, preMS samples, and pwMS. (**B**) Box plots of Tfh-to-Tfr ratio in the mantle zone and follicle in controls, preMS, and pwMS. (**C**) Spatial interactions between Tfh cells and BCL6^+/–^ GC B cells in follicles with active GCs using box plots. Box plots of B cell subsets between pwMS and controls. Box plots show the interquartile range, median (line), and minimum and maximum (whiskers). In all, the BH-corrected unpaired Wilcoxon test was used and *P* values < 0.05 are shown.

Spatial compartmentalization revealed further distinctions. Tfh-like cells were increased in the mantle zone (*P_adj_* = 0.043), whereas Tfh cells were elevated in the follicle (*P_adj_* = 0.041) in preMS compared with controls (Figure 5A). While we did not see a difference in the relative positioning of Tfh or Tfr cells in preMS samples using the follicle-mantle ratio, the Tfh-to-Tfr ratio was elevated in the mantle zone of preMS LNs (*P_adj_* = 0.014). This suggests that the regulation of Tfh cells is decreased before MS diagnosis, similar to what was seen in pwMS (Figure 5B).

Finally, we performed spatial proximity analysis for samples with GCs. Tfh had increased interaction with both BCL6^+^ (*P_adj_* = 0.029) and BCL6^–^ GC B cells (*P_adj_* = 0.018) in preMS samples compared with controls (Figure 5C), while interactions of Tfh cells with naive B cells or Tfr cells in the mantle zone or follicle were similar. Additionally, Tfh-like cells had increased interactions with BCL6^–^ GC B cells in preMS (Supplemental Figure 5D).

Together, these results indicate that Tfh cell expansion and altered spatial interactions with B cells are already evident in LNs before MS diagnosis, supporting immune dysregulation in LNs as an early event in MS pathogenesis.

### EBV latency not detected in LNs of pwMS or controls.

**Figure 6:**
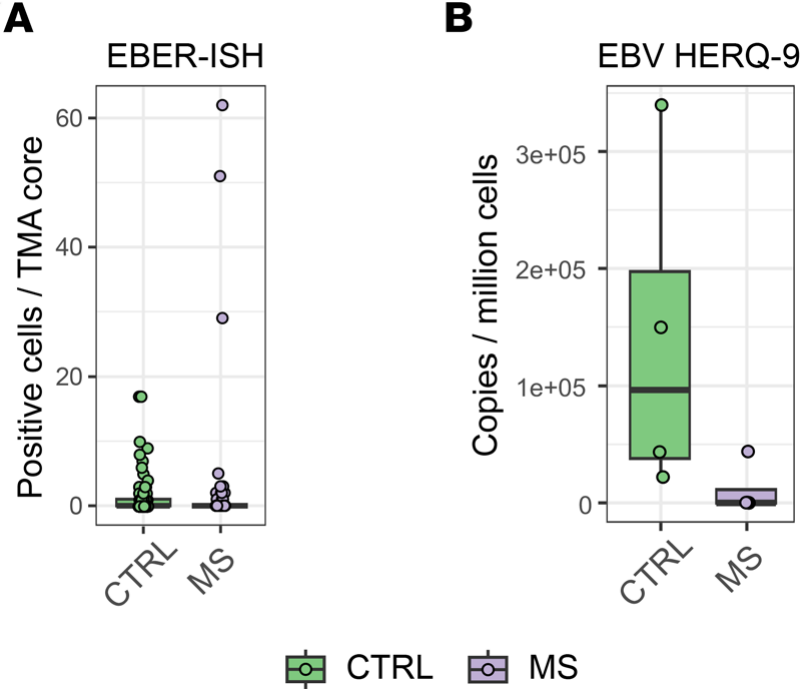
Detection of EBV in FFPE LNs. (**A**) Box plot of EBER-positive cells in each TMA core between pwMS and controls. (**B**) Box plot of EBV copies per million cells between pwMS and controls using HERQ-9 multiplexed qPCR. For this, tissue sections from each TMA block were divided into 2 pieces. Box plots of B cell subsets between pwMS and controls. Box plots show the interquartile range, median (line), and minimum and maximum (whiskers). The BH-corrected unpaired Wilcoxon test was used in **A** and **B**.

As we recently detected alterations in GC reaction together with EBV-driven B cell dysregulation in dcLNs in early MS (12), we were interested in whether latent EBV infection could be driving the detected immune alterations in LNs of pwMS. The EBER-ISH test is a diagnostic technique used to detect EBV infection by identifying the presence of EBV-encoded small RNAs (EBER1 and EBER2) in tissue samples. These RNAs are highly expressed in B cells latently infected with EBV, making them reliable markers for the virus (28). We thus performed EBER-ISH on the constructed TMA sections. Both MS and control samples were largely negative (Figure 6A and Supplemental Figure 6A). Three pwMS samples showed higher numbers of EBER-positive cells than controls, but these individuals did not differ in clinical characteristics. The preMS samples had similar numbers of EBER-positive cells as pwMS and controls.

We next applied multiplex quantitative PCR (HERQ-9) (29) to the TMA sections to detect EBV and other common herpesviruses (Supplemental Figure 6B). EBV DNA was again only sporadically detected, with no significant differences between MS and control groups (Figure 6B). HHV-6B and HHV-7 were also detected, with HHV-7 more frequently observed in controls (Supplemental Figure 6C). All other herpesviruses tested negative. Thus, no major differences in herpesvirus latency were detected in FFPE LNs between patients and controls.

## Discussion

For pwMS, circulating T and B cells of the peripheral blood and CSF have been described in depth, whereas the essential immune programming milieu of the lymphoid tissues has remained mainly uncharted. This study investigated FFPE LN samples from pwMS and controls using highly multiplexed imaging. We characterized nearly 2.4 million single cells and found an expansion of follicular T cells in pwMS. Compared with controls, Tfh cells were more prevalent in the mantle zone, outside the GC, than their regulatory counterparts. Additionally, we observed increased spatial interactions between Tfh cells and FoB cells in the LNs of pwMS. The expansion of Tfh cells was evident even in LNs collected before the diagnosis of MS, implying a role in disease pathogenesis.

Tfh cell development is a multifaceted process, beginning with naive T cells being directed to the Tfh lineage by DCs, followed by migration of pre-Tfh cells to the mantle zone (13). The accumulation of Tfh cells in the mantle zone (or in the T-B border) could explain the increased circulatory Tfh cells reported by several studies (15, 18, 30), as Tfh cells in circulation originate from the mantle zone of SLOs (31, 32). Additionally, the abundant PD-1^+^ Tfr cells in the follicle possibly represent a population recently developed from the Tfh pool (33), due to the expansion of Tfh cells. Interestingly, we have observed a similar accumulation of Tfh-like (or pre-Tfh) cells in dcLNs in MS (12) but also in patients with inborn errors of immunity, such as autoimmune polyendocrinopathy–candidiasis–ectodermal dystrophy and those with mutations in *IKZF2* (encoding transcription factor Helios) (26, 34). Tfh cell fates in the mantle zone warrant further investigations, as prolonged activation of Tfh cells can promote unspecific B cell activation (35), and Tfh cells are increased in circulation in several other autoimmune diseases (36–39).

Excess Tfh help can lower the need for T-B interactions, allowing B cells with various specificities to develop into effector B cells (40). We observed increased interactions between BCL6^+/–^ FoB cells and Tfh cells and modest interaction with Tfh-like cells in pwMS. Also, interactions with Tfh cells and naive B cells were increased in the mantle zone. The increased interactions between Tfh and B cells, especially at the mantle zone where B cell fate is determined (14), could represent an excess of Tfh help, allowing for the selection of autoreactive B cells. Our results align with our recent study, where we detected increased Tfh-like cells in dcLNs of patients with early MS (12). On the other hand, in that study, the GC B cells and GC-Tfh cells were diminished, suggesting an impaired GC reaction. While cyclic multiplex imaging benefits from simultaneous protein expression and spatial location in cell profiling, the number of markers is limited, and identification of Tfh subsets is not as detailed as with single-cell RNA sequencing. Additionally, our mfIHC panel was focused on follicular T cells, and the in-depth characterization of B cells was beyond the scope of this study.

In a post–acute infection setting, EBV proteins have been found in the cervical LN of a patient with MS but not in other LN locations, which aligns with our results here (41). Alternatively, EBV may not be replicated at the chronic stage of the disease, as the median duration of MS in our cohort was 15 years. In our prior study, we detected EBV and EBV-targeting Tc cells in early-MS dcLNs (12). Therefore, the presence of EBV should be investigated from fresh LNs in chronic MS, as detection of viral DNA, including EBV, is more sensitive in fresh than in FFPE tissues (42). EBV is causatively connected with MS development (2), and persistent viral infections, such as those caused by EBV, can increase Tfh cells and drive polyclonal B cell responses (43, 44). Thus, chronic local inflammation by EBV in early MS may support Tfh cell accumulation outside the follicles and stimulate B cell–mediated presentation of self-peptides or EBV antigens to autoreactive CD4^+^ T cells, promoting autoimmunity (45).

Our study has limitations. First, the samples were retrieved from the Helsinki Biobank, typically collected during clinical evaluation of suspected malignancies. While we matched the controls for several factors, including age, sex, anatomical location, and possible cancer, we could not study the possible effect of recent infections or vaccines. Also, the patients had different stages of MS upon removal of LNs. Therefore, other variables may also contribute to the differences observed in Tfh cells between pwMS and controls. Second, the LNs were from several anatomical locations and contained GCs in varying numbers. This heterogeneity may affect the results, especially regarding bona fide Tfh cells and specialized B cells. Moreover, dcLNs were not well represented in our cohort; however, our findings on elevated Tfh cells in pwMS appeared independent of both anatomical location and cancer presence.

In conclusion, our findings highlight LNs as a site of an early and persistent alteration in immune activation in pwMS. Tfh excess help could be a key mechanism of autoreactive B cell maturation in MS, further leading to activation of CNS-targeting T cells. Whether EBV infection has a role in the origin of the Tfh alterations remains to be further investigated, as well as the potential therapeutic implications of our findings.

## Methods

### Sex as a biological variable.

Our study examined FFPE human LNs in both men and women, and similar findings are reported for both sexes. Therefore, the findings are expected to be relevant to both males and females, even though most LNs were from females.

### Study design.

From the Helsinki Biobank, we requested all FFPE LN samples from pwMS (ICD-10 diagnosis G35), and for control, LN samples without MS, other demyelinating diseases of CNS (G37.9), neoplasms (C00-D48), or systemic connective tissue disease (M30-36) (Figure 1A). The LNs were removed because of clinical reasons at Helsinki University Hospital between the years 2008 and 2019 and were initially reviewed by a pathologist and declared free of cancer. From the LNs resulting from 103 pwMS (MS LN) and 102 controls (control LN), the single most representative H&E-stained section from every sample was retrospectively digitized by the Helsinki Biobank (Pannoramic 250 FLASH III scanner, 3DHISTECH) with a 20× objective. A pathologist reevaluated the digitized sections and histopathological diagnoses. From each LN, 2 B cell–rich follicular areas were annotated by morphology (Supplemental Figure 1A) using 1.5 mm diameter circles, based on which TMAs were constructed in collaboration with the Helsinki Biobank. Samples without visual B cell follicles were excluded from the analysis. In total, 102 MS and 172 control LN punches from 61 pwMS and 86 controls were accepted for viral assays and single-cell–resolution multiplex imaging. For the MS LNs chosen for TMA, we confirmed MS diagnoses and collected individual clinical data using the electronic healthcare records of Helsinki University Hospital.

### EBER-ISH.

EBER-ISH immunostainings were performed using standard protocols at the Department of Pathology, Helsinki University Hospital. Helsinki Biobank did the chromogenic scanning using a 3DHISTECH Pannoramic 250 FLASH II digital slide scanner. Data from EBER-ISH were analyzed in QuPath (46) (version 0.4.3) using the “Positive cell detection” tool.

### DNA extraction of the TMA samples.

Each TMA was divided into 2 parts for DNA extraction, resulting in 4 MS and 4 control samples. Five consecutive 5 μm–thick sections were cut and processed from each sample using a QIAamp DNA FFPE Advanced extraction kit (QIAGEN catalog 56604). Each section was extracted according to the manufacturer’s protocol. The extracted DNA was eluted in 60 μL of ATE buffer and stored at –20°C.

### Real-time qPCR (HERQ-9 and RNaseP).

The extracted DNA was analyzed for all 9 human herpesviruses (HSV-1, HSV-2, VZV, EBV, HCMV, HHV-6A, HHV-6B, HHV-7, and KSHV) by multiplex qPCR assays according to Pyöriä et al. (29). The amplifications and quantifications of viral DNA were performed with the AriaMx Real-Time PCR System (Agilent). For normalization of the viral DNA and as a positive control, the single-copy human gene RNaseP was quantified as previously described (47). DNA extractions and handling of samples and qPCR reagents were performed in separate rooms and hoods to prevent contamination.

### Characteristics of the patient cohort for single-cell spatial analysis.

We identified samples with CD20 and CD21 staining patterns typical of B cell follicles and the quality control steps (Supplemental Methods). Subsequently, our single-cell spatial data cohort consisted of 28 pwMS, 5 preMS, and 35 non-MS controls (Table 1 and Supplemental Table 1). The control LNs were matched for age, sex, anatomical location, and possible cancer. For most individuals, LNs were removed because of local malignancy (pwMS: *n* = 19, 58%; controls: *n* = 18, 51%). However, in some cases, metastatic activity was observed in sLNs (pwMS: *n* = 11, 33%; controls: *n* = 12, 34%) but not in the LNs chosen for this study. At the time of sample collection, roughly half the patients had immunosuppressive medication, including interferon-β, azathioprine, glatiramer acetate, dimethyl fumarate, or alemtuzumab. The disability caused by MS was assessed using the EDSS (48) and compared between the time of diagnosis and the last visit at the neurology outpatient clinic in the Helsinki University Hospital using the electronic healthcare records.

### mfIHC staining and image processing.

mfIHC staining and imaging of the TMA sections were done using 6 staining cycles by the FIMM Digital Microscopy and Molecular Pathology Unit (University of Helsinki), as described earlier (26, 49). Briefly, 4 μm–thick TMA sections were first stained with fluorescent antibodies supplemented with DAPI stain following high-resolution imaging, after which the fluorescent signal was bleached for 1 hour at room temperature and denatured for 20 minutes in 99°C Tris/EDTA (pH 9) buffer. Antibodies and incubation times are shown in Supplemental Table 3.

Whole slide imaging was performed using a ZEISS Axio Scan.Z1 for each cycle. Image processing was done as explained previously (26), which is explained in detail in the Supplemental Methods.

### Image analysis.

Nuclei segmentation using StarDist (50), MFI calculations for each cell, and quality control steps were performed, as in our previous study (Supplemental Methods) (26). The quantified single-cell data from the follicular image crops were normalized (Supplemental Table 4), phenotyped, and analyzed using Scimap (version 1.3.1; Supplemental Figure 1, B–D, and Supplemental Table 5) (51). The positive thresholds were set manually for each TMA, after which the signal intensities of the cropped LN images were normalized for each TMA separately.

Due to incomplete bleaching of the cytoplasmic CD27 signal from the previous staining cycle, nuclear BCL6 staining was partially obscured. To minimize cytoplasmic signal contamination, the BCL6 expression was quantified specifically within nuclear masks that were uniformly shrunk by 2 pixels. To account for variability in raw intensity values across samples — arising from both biological differences and technical factors such as staining efficiency or imaging conditions — we standardized BCL6 expression levels by binning continuous intensity values. This was achieved by independently applying a 5-component Gaussian Mixture Model (GMM) to each sample’s intensity distribution. The GMM probabilistically separated cells into 5 expression states, ranked by their mean intensity values and labeled as background, negative, low, medium, or high. Based on visual inspection of the original images, cells classified in the “high” expression group were considered BCL6 positive and used in the downstream analyses (Supplemental Figure 1E).

Images containing over 30% of “Unknown” cells were excluded. If multiple follicular images from an individual fulfilled the quality control criteria, the image with the lowest frequency of “Unknown” cells was chosen. After quality control steps, single-cell image data from 33 pwMS and 35 controls were accepted for further analysis (1 follicular image per individual; Supplemental Table 1). From these, double-positive (CD3^+^CD4^+^CD8^+^) T cells (38 cells, 0.002%) were removed from further analysis because of low cell counts together with cells with autofluorescence typical for red blood cells (99,815 artifact cells, 4.0%).

### Single-cell spatial analysis.

The neighborhood analyses were adopted from Scimap using Jupyter Notebook with Python (version 3.9.16). The average shortest distance between each cell type was calculated to study cellular distances across the samples. Cell neighborhoods were investigated by comparing how likely 2 given cell types are to be found next to each other compared with the random background using the K-nearest neighbors method with the 10 nearest cells to determine the neighborhood for every cell (51, 52).

### Immunohistochemistry.

BCL6, PD-1, and Ki67 (MIB-1) immunostainings were performed using standard protocols at the Department of Pathology, Helsinki University Hospital (Supplemental Table 2). The chromogenic scanning was done by Helsinki Biobank using 3DHISTECH Pannoramic 250 FLASH II digital slide scanner. Follicle crops were divided into primary follicles without active GCs and secondary follicles with active GCs based on the staining pattern of BCL6, PD-1, and Ki67 typical for GCs (Supplemental Figure 2B).

### Statistics.

An unpaired Wilcoxon’s test with BH adjustment (*P_adj_*) was used to compare the 2 categories. *P_adj_* of less than 0.05 was considered significant. *P* values for the cell type–specific cellular interactions were calculated by subtracting the permuted mean from the observed mean divided by the number of permutations (27) or using an unpaired Wilcoxon’s test to compare the cell-cell spatial interaction of given cell types between 2 categorical groups (26). All calculations were done with R (4.3.1) or Python (3.9.16).

### Study approval.

The Helsinki University Hospital ethical committee and the Helsinki Biobank approved this study (decision number HUS/2123/2019; research permit number HUS/466/2019).

### Data availability.

The multiplex fluorescence images that support the findings of this study are available at Synapse with login at https://doi.org/10.7303/syn63635802

Values for all data points in the figures are reported in the Supporting Data Values file.

The codes to reproduce the key findings are available on GitHub (https://github.com/SarkkinenJ/MS_GC_image_analysis.git; commit ID 24b045e).

## Author contributions

JS, EK, MIM, and SML designed the study. JS, JD, and SML acquired the data. JS, JD, LH, AJ, and MFP conducted the experiments, and JS, AJ, and LH analyzed the data. EK, MFP, MIM, and SML provided reagents. JS, EK, and SML wrote the manuscript. JS, EK, LH, AJ, JD, MFP, MIM, and SML commented on and edited the manuscript.

## Supplementary Material

Supplemental data

Supplemental tables 1-5

Supporting data values

## Figures and Tables

**Table 1:**
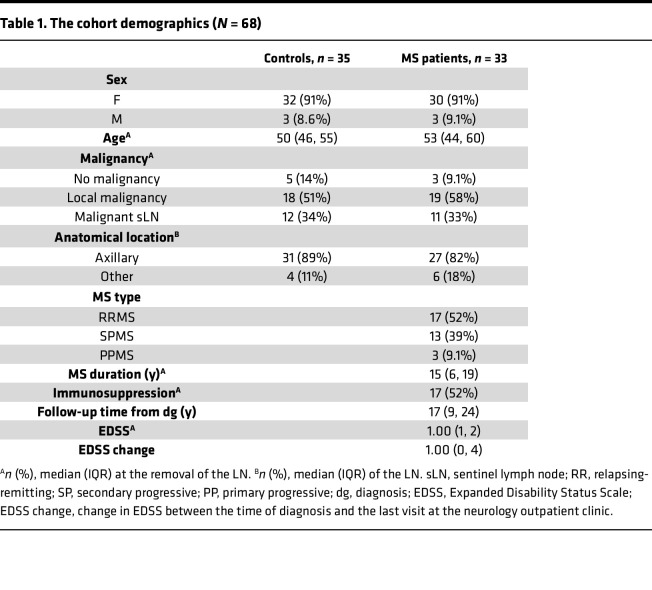
The cohort demographics (*N* = 68)

## Version 1 10/08/2025

Electronic publication
