# Supplementary material for: Intranodal expansion of follicular T helper cells in patients with multiple sclerosis
Source: JCI Insight. 2025 Oct 8;10(19):e188125. doi: 10.1172/jci.insight.188125 (PMC12513493; doi:10.1172/jci.insight.188125)
Supplement: Supplemental data [file jciinsight-10-188125-s034.pdf]

Supplemental Materials for "Intranodal Expansion of Follicular T Helper cells in Patients with Multiple Sclerosis" by Sarkkinen *et al*.

Table of contents:

- Supplemental Materials on methods

- Supplemental Figures 1-6

Supplementary Methods

Image processing

Whole slide imaging was performed using a Zeiss Axio Scan.Z1 with Zeiss Plan-Apochromat objective 20x (0.8NA, M27), Hamamatsu ORCA-Flash 4.0 V2 Digital CMOS camera (16-bit; 0.325 µm/pixel resolution), and Zeiss Colibri.7 LED Light Source. Filter specifications were DAPI cube (Zeiss Filter Set 02), FITC cube (Zeiss Filter Set 38 HE), Cy3 cube (Chroma Technology Corp 49004 ET CY3/R), Cy5 cube (Chroma Technology Corp 49006 ET CY5), and Cy7 cube (Chroma Technology Corp 49007 ET CY7). Images were exported as whole-slide 16-bit TIFF single-channel grayscale images with 25% and 100%.

Cropping of TMA cores was adopted from Pellinen et al. (Pellinen *et al.*, 2022). In short, cores with CD20 and CD21 staining patterns typical for B cell follicles were identified and subsequently annotated with 1500 x 1500 pixels rectangles (1 pixel = 1 µm) using Roi1 1-Click Tools macro in Fiji (version 2.3.0) and recorded as one CSV file per whole slide using ROI Manager List tool. ROI annotations were performed on 25% resolution DAPI images, and the resulting CSV files were also applied to 100% images using a scaling factor of 4. MATLAB (version R2019b, MathWorks Inc, Natick, MA, USA) was used to crop the grayscale cores from the whole slide TMA images based on the annotations mentioned above, after which crops from each cycle were aligned with each other based on DAPI signal using an in-house script. Image stacks were subsequently created with CellProfiler (version 4.0.5) using the "GrayToColor" module.

Image analysis

To attain a single-cell level, nuclei were segmented using the first-round nuclei image using StarDist (Schmidt *et al.*, 2018). The trained Versatile (fluorescent nuclei) model was further trained using ZeroCostDL4Mic Colab (von Chamier *et al.*, 2020), where annotated cells from the tertiary lymphoid structure dataset (Sarkkinen *et al.*, 2023) were used as ground truth, whereas training was performed based on corresponding nuclei images applied with Gaussian Blur to mimic highly cell-rich follicles. Nuclei images were also used to detect and calculate tissue area in each crop using the "Thresholder"-tool in QuPath (Bankhead *et al.*, 2017) (version 0.4.3).

The median fluorescence intensity (MFI) for each cell mask was computed using the Bio-Formats library in Matlab. Cells which had moved or washed away during the cyclic staining protocol were filtered using the repeated DNA MFI measurement as a reference as was performed (Launonen *et al.*, 2022; Hetemäki *et al.*, 2023). As a part of quality control, we also compared MFIs in each image crop to the age of the FFPE sample and observed that the staining patterns were somewhat similar throughout the samples (**Supplementary Figure 1E**).

Bankhead, P. *et al.* (2017) 'QuPath: Open source software for digital pathology image analysis', *Scientific reports*, 7(1), p. 16878.

von Chamier, L. *et al.* (2020) 'ZeroCostDL4Mic: an open platform to simplify access and use of Deep-Learning in Microscopy', *bioRxiv*. Available at: https://doi.org/10.1101/2020.03.20.000133.

Hetemäki, I. *et al.* (2023) 'Dysregulated germinal centre reaction with expanded T follicular helper cells in APECED lymph nodes', *The Journal of allergy and clinical immunology* [Preprint]. Available at: https://doi.org/10.1016/j.jaci.2023.12.004.

Launonen, I.-M. *et al.* (2022) 'Single-cell tumor-immune microenvironment of BRCA1/2 mutated high-grade serous ovarian cancer', *Nature communications*, 13(1), pp. 1–14.

Pellinen, T. *et al.* (2022) 'Fibroblast subsets in non-small cell lung cancer: associations with survival, mutations, and immune features', *Journal of the National Cancer Institute* [Preprint]. Available at: https://doi.org/10.1093/jnci/djac178.

Sarkkinen, J. *et al.* (2023) 'Single-cell spatial atlas of tertiary lymphoid structures in ovarian cancer', *bioRxiv*. Available at: https://doi.org/10.1101/2023.05.16.540946.

Schmidt, U. *et al.* (2018) 'Cell Detection with Star-Convex Polygons', in *Medical Image Computing and Computer Assisted Intervention – MICCAI 2018*. Springer International Publishing, pp. 265–273.

Supplementary Figure 1

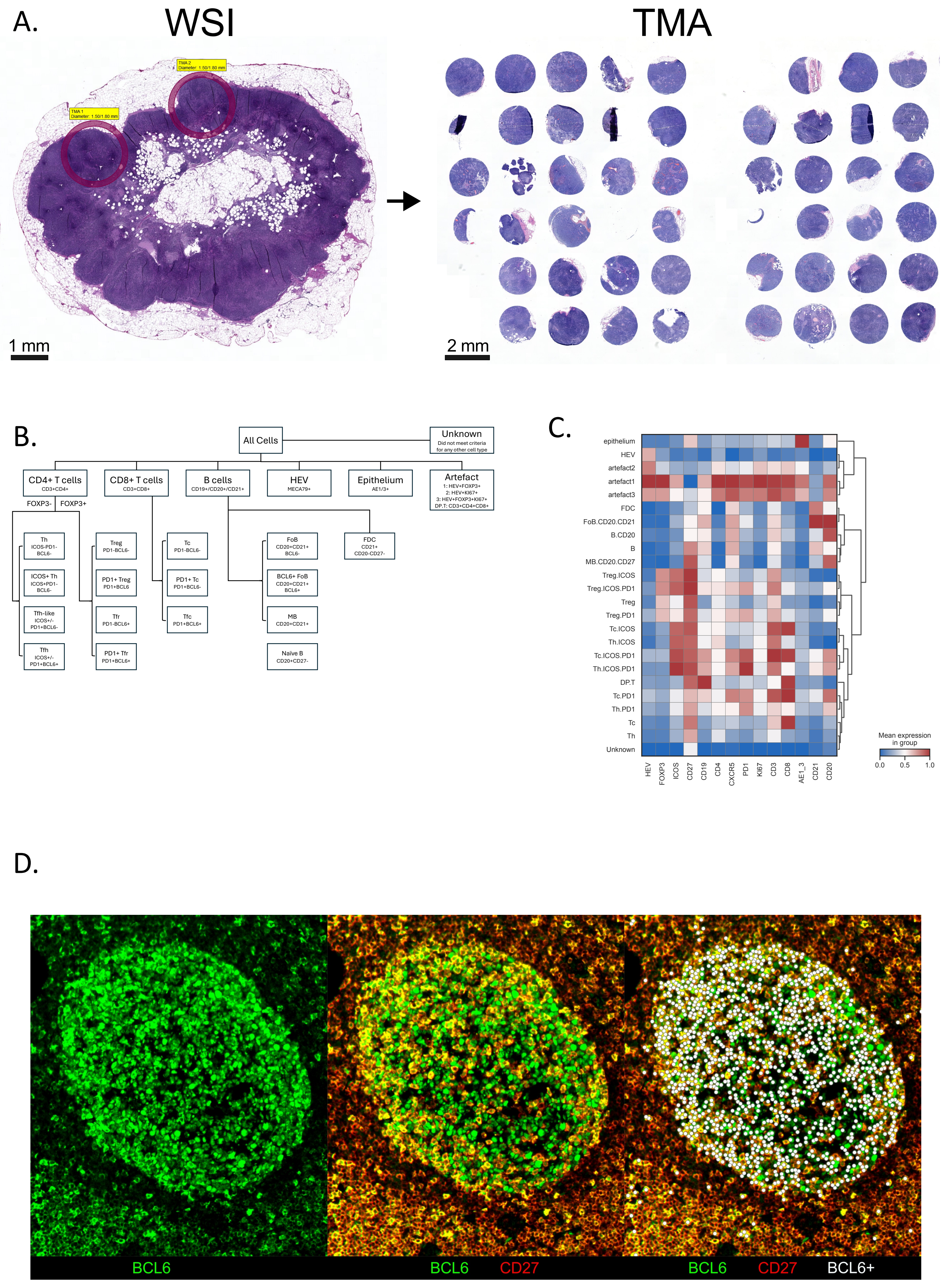

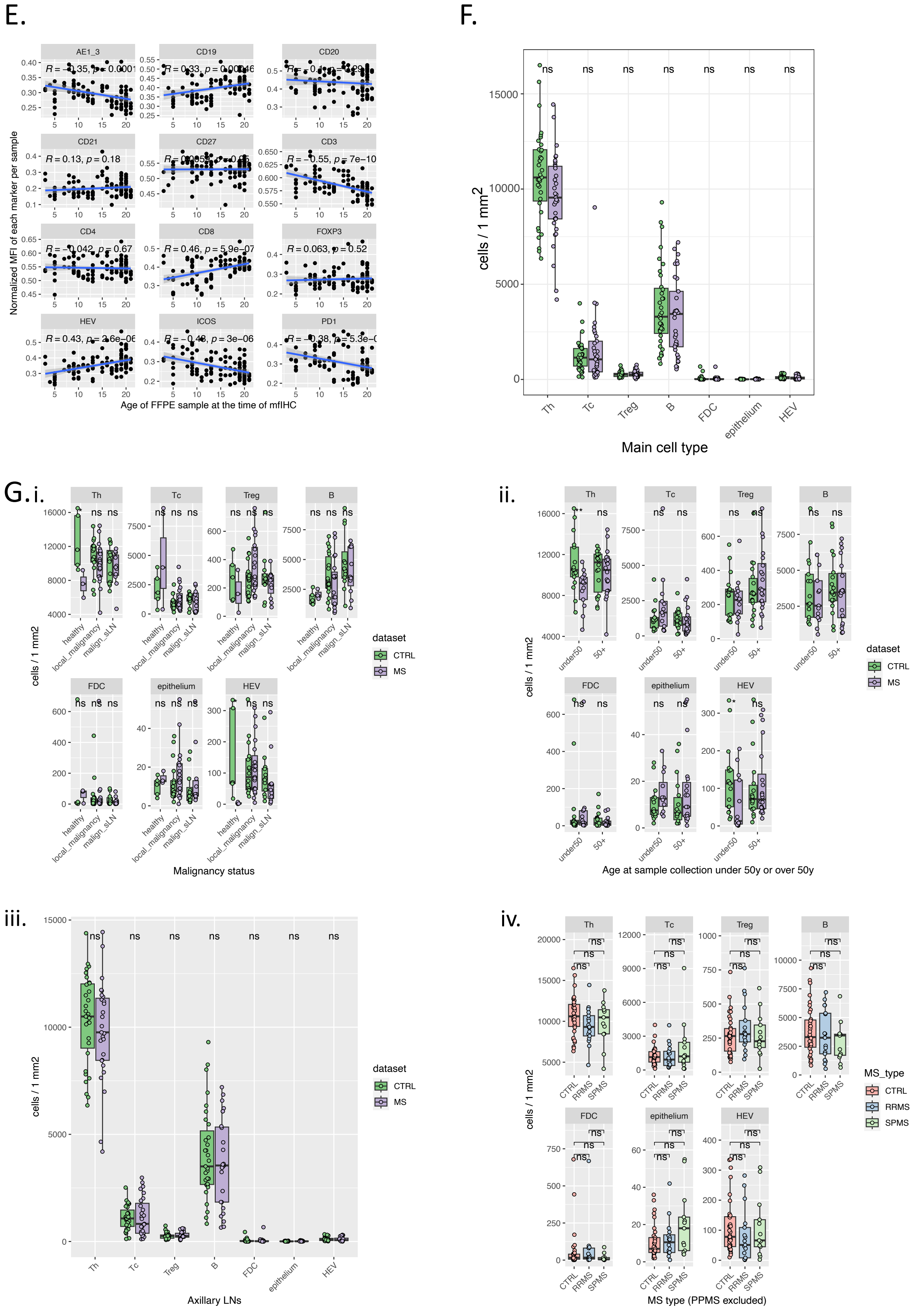
E.
AE1_3
CD19
CD20
CD21
R = 0.13, p = 0.18
CD27
CD3
R = −0.55, p = 7e−10
CD4
CD8
R = 0.46, p = 5.9e−07
FOXP3
R = 0.063, p = 0.52
HEV
ICOS
R = −0.48, p = 3e−06
PD1
Normalized MFI of each marker per sample
Age of FFPE sample at the time of mfIHC
F.
cells / 1 mm2
Main cell type
Th
Tc
Treg
B
FDC
epithelium
HEV
ns
G.i.
dataset
CTRL
MS
healthy
local_malignancy
malign_sLN
Malignancy status
ii.
under50
50+
Age at sample collection under 50y or over 50y
iii.
Axillary LNs
iv.
MS_type
CTRL
RRMS
SPMS
MS type (PPMS excluded)

v.

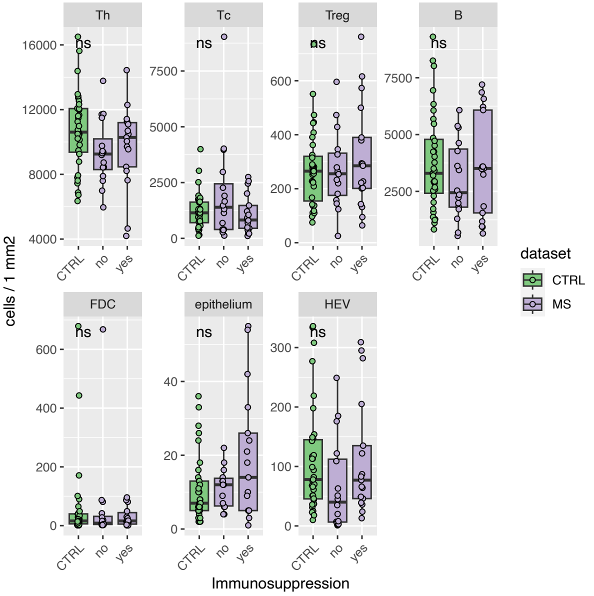

vi.

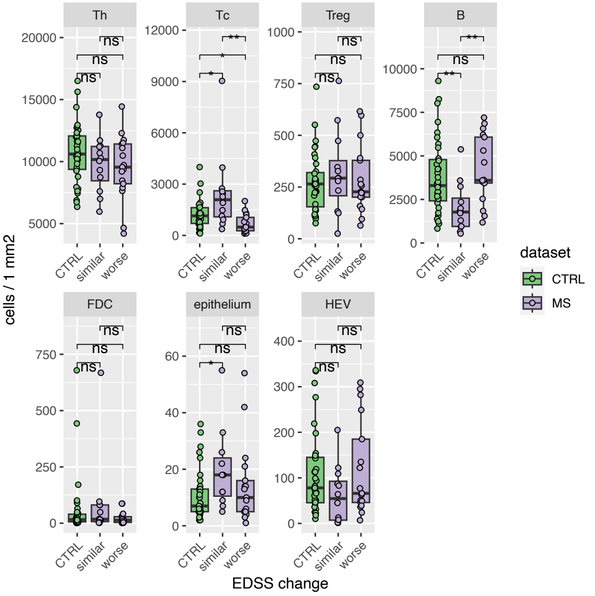

Supplementary Figure 1: Landscape of single cells in lymph node images of pwMS and controls. (A) An illustration with scale bars showing how ROIs and tissue microarrays were constructed. **(B)** A diagram demonstrating hierarchical cell phenotyping. **(C)** Heat map with dendrogram showing correlations of scaled marker expressions in each cell type. **(D)** Fluorescent images showing nuclear BCL6 staining together with incompletely bleached cytoplasmic CD27 staining from the previous staining cycle. Left: nuclear (true) and cytoplasmic staining (from CD27) in the BCL6 channel. Middle: the cytoplasmic staining in the BCL6 channel is derived from cytoplasmic CD27 (red/orange), while the true BCL6 signal (green) is detectable in the nuclei. Right: BCL6-positive cells (white dots) were identified from shrunken nuclear masks using the Gaussian Mixture Model. **(E)** Scatter plots with Pearson correlations between the age of the FFPE samples and normalised MFIs. **(F)** Box plot of main cell types in LN follicles between pwMS and controls. **(G)** Box plots of main cell subsets compare i) possible malignancy in the individual, ii) age at removal of the lymph node, iii) axillary lymph nodes, iv) different types of MS, v) possible immunosuppressive medication, and vi) change in Expanded Disability Status Scale (EDSS) in pwMS and controls. A Wilcoxon test was used to calculate *P*-values.

Supplementary Figure 2

A.

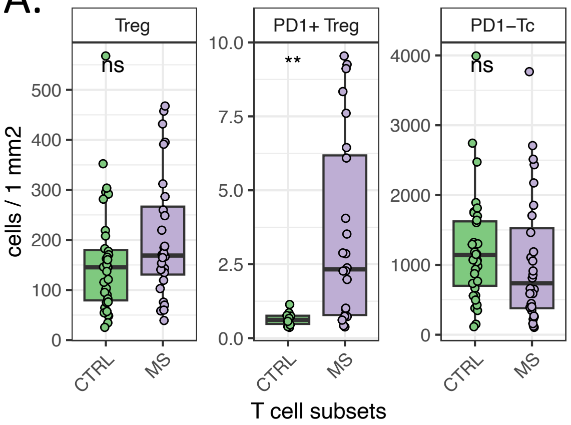

B.

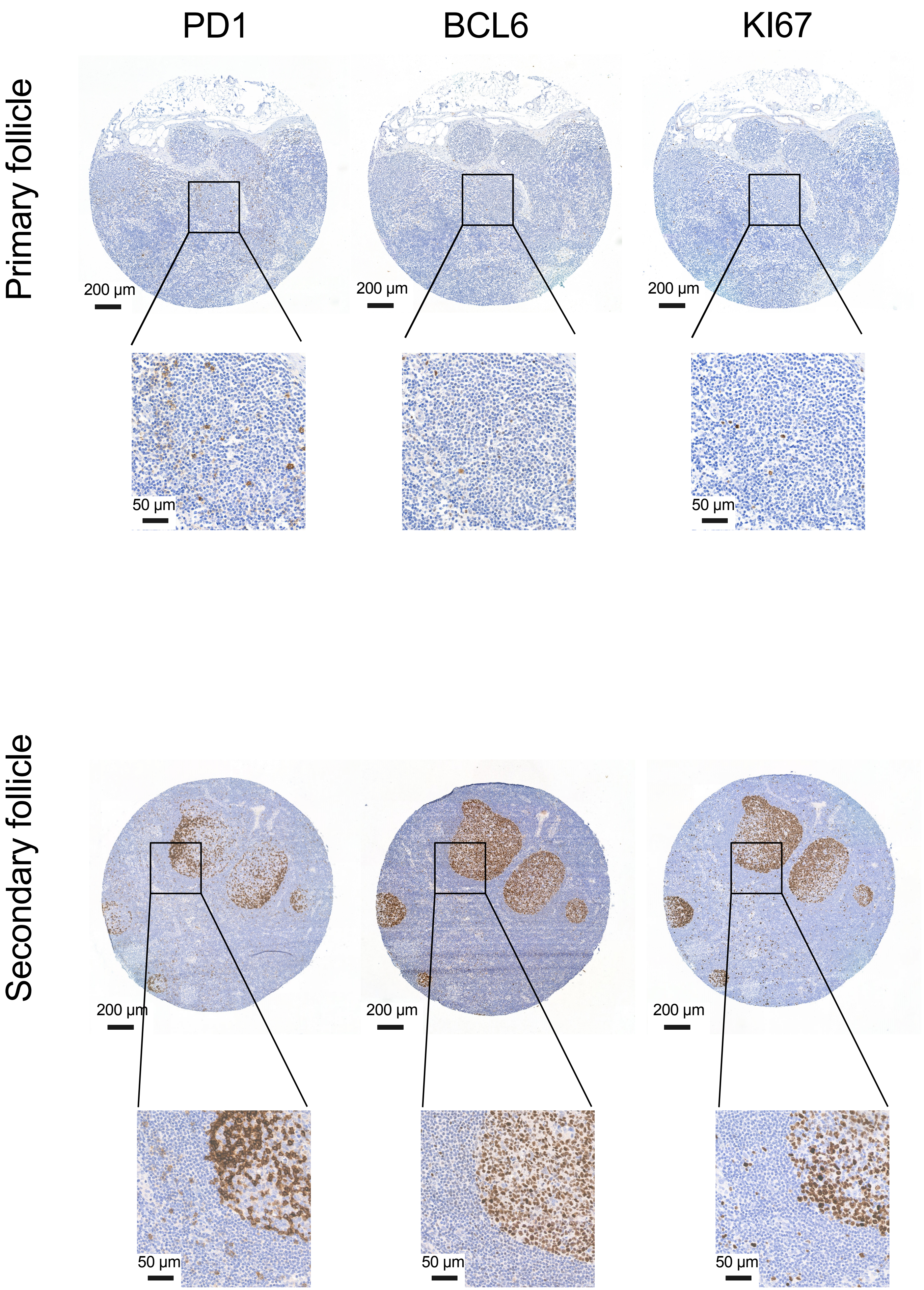
PD1
BCL6
KI67
Primary follicle
Secondary follicle
200 µm
50 µm

C.

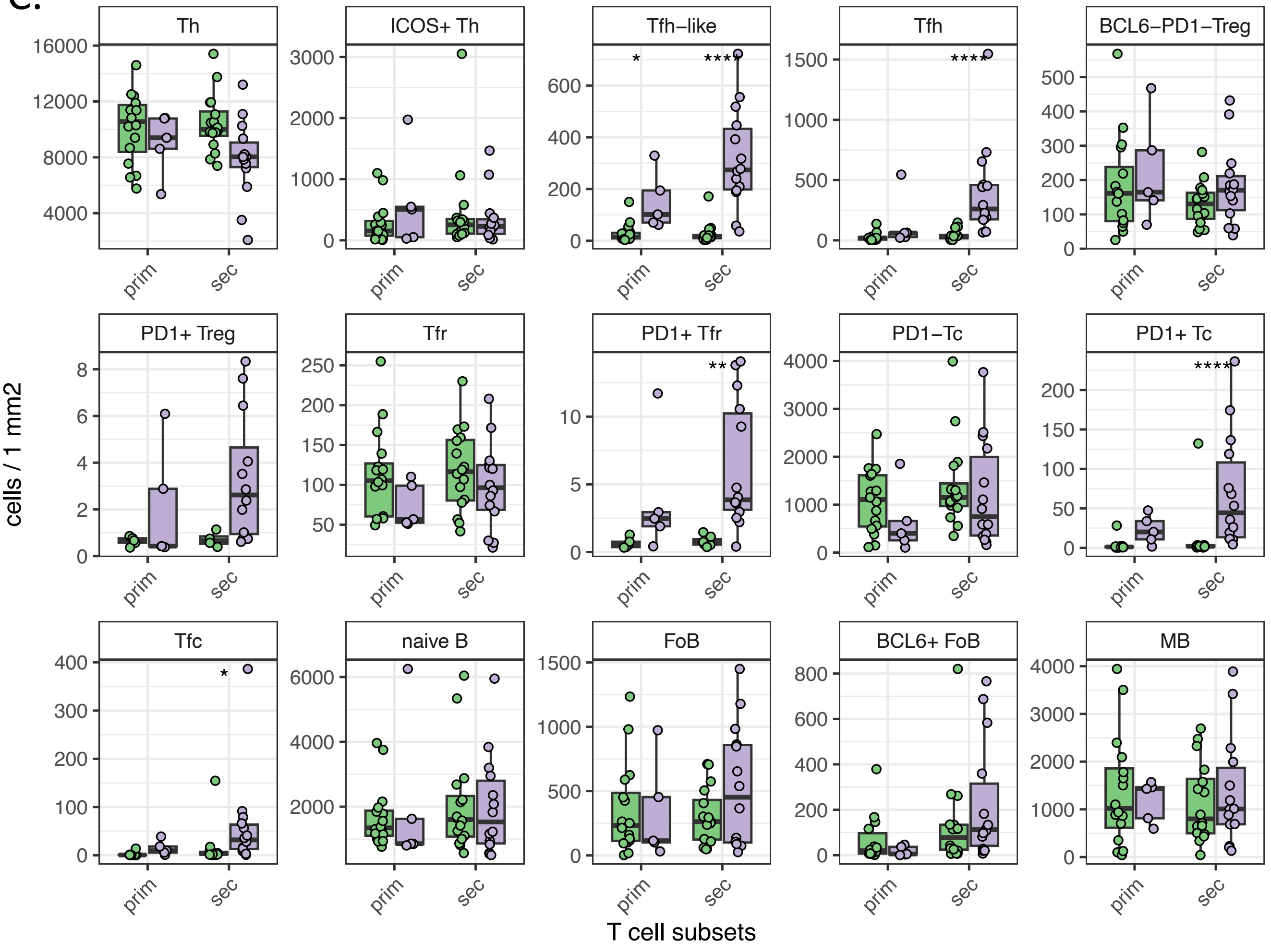
Th
ICOS+ Th
Tfh−like
Tfh
BCL6−PD1−Treg
PD1+ Treg
Tfr
PD1+ Tfr
PD1−Tc
PD1+ Tc
Tfc
naive B
FoB
BCL6+ FoB
MB
prim
sec
cells / 1 mm2
T cell subsets

D.

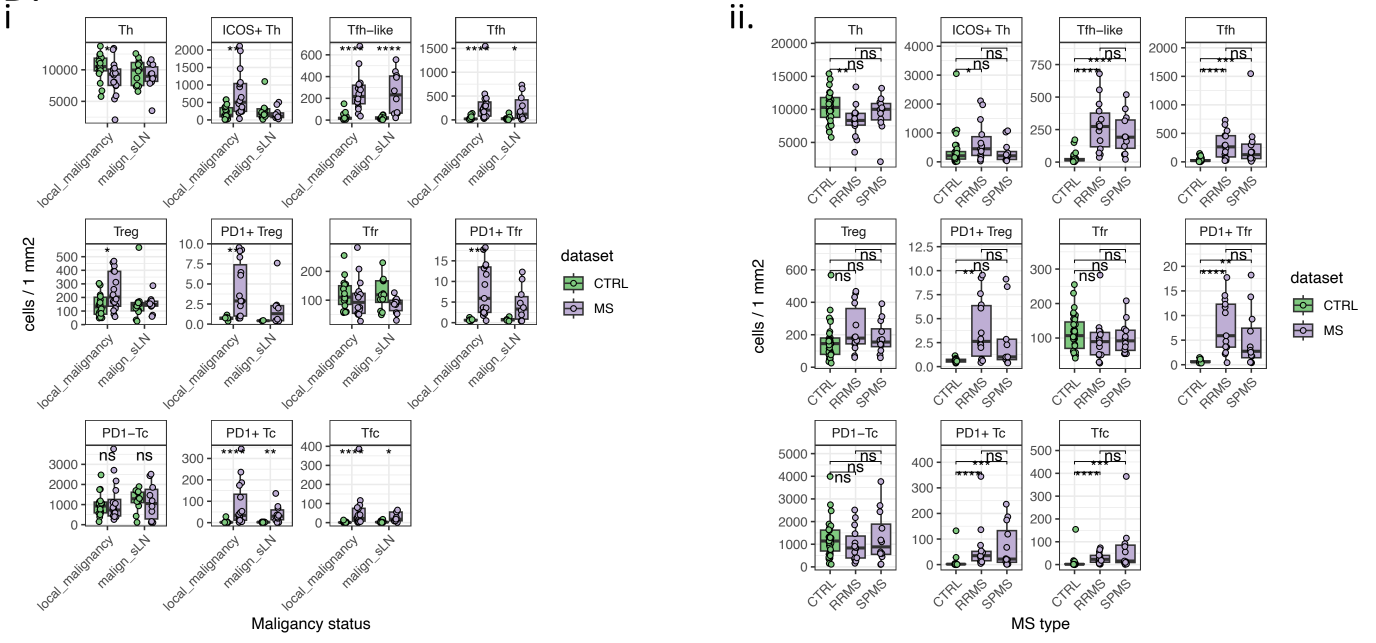
i
ii.
Th
ICOS+ Th
Tfh−like
Tfh
Treg
PD1+ Treg
Tfr
PD1+ Tfr
PD1−Tc
PD1+ Tc
Tfc
local_malignancy
malign_sLN
cells / 1 mm2
Maligancy status
dataset
CTRL
MS
CTRL
RRMS
SPMS
MS type

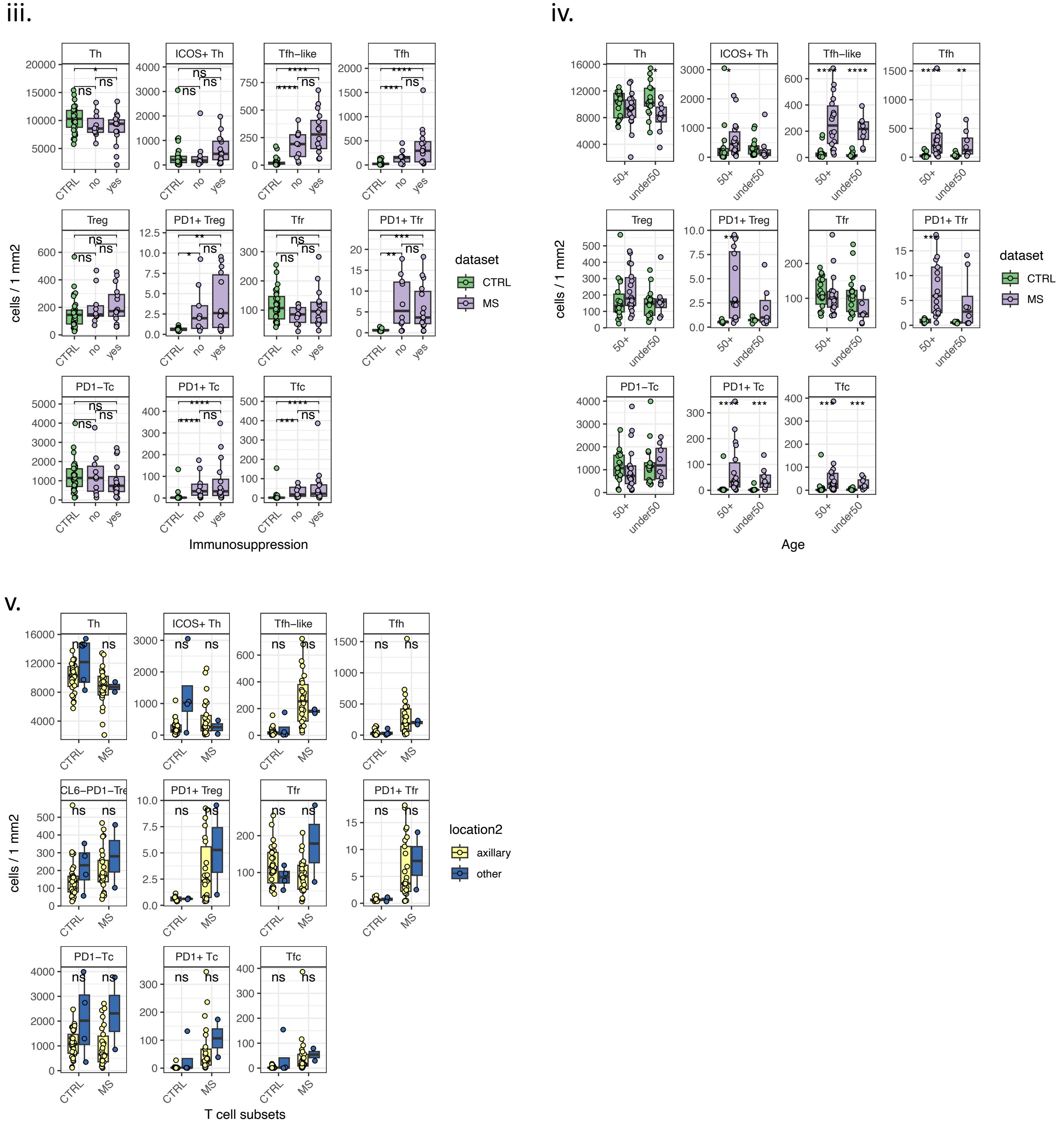

Supplementary Figure 2: Lymphocyte subsets in lymph nodes of pwMS and controls. (A) Box plots of T cell subset numbers that are not shown in Figure 2B. **(B)** IHC images with scale bars and magnifications of PD1 (left), BCL6 (middle) and KI67 (MIB-1, right) stainings. The images show examples of samples with primary (top) and secondary follicles (bottom). **(C)** Box plots of T and B cell subsets in primary and secondary follicles between pwMS and controls **(D)** Box plots of T cell subsets compare i) possible malignancy in the individual, ii) different types of MS, iii) possible immunosuppressive medication, iv) age at removal of the lymph node, and v) axillary lymph nodes to lymph nodes from other anatomical sites in pwMS and controls. The unpaired Wilcoxon test was used in A, C-D. BH-corrected p-values are shown in A and C. Unsignificant values are not shown, * P < 0.05, ** P < 0.01, *** P < 0.001, and **** P < 0.0001.

Supplementary Figure 3

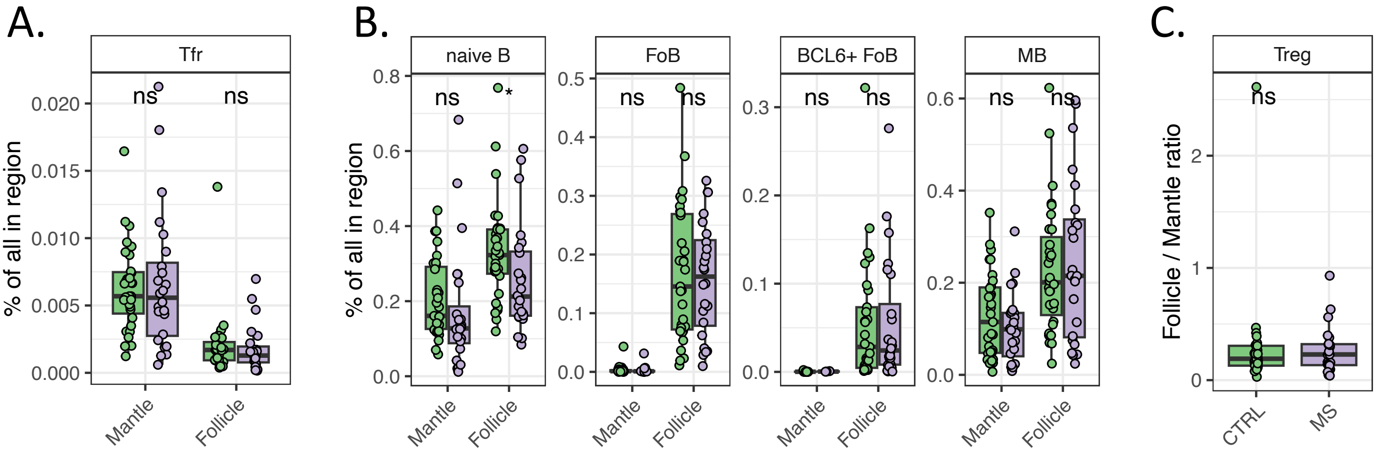

Supplementary Figure 3: Proportions of lymphocyte subsets in the mantle zones and follicles. (A-B) Box plots of (A) Tfr cell and (B) B cell subset frequencies in the mantle zone and follicle. **(C**) The follicle-mantle zone ratio of all FOXP3+ Treg cells using box plot. The unpaired Wilcoxon test was used with BH-correction. P < 0.05 is highlighted with *.

Supplementary Figure 4

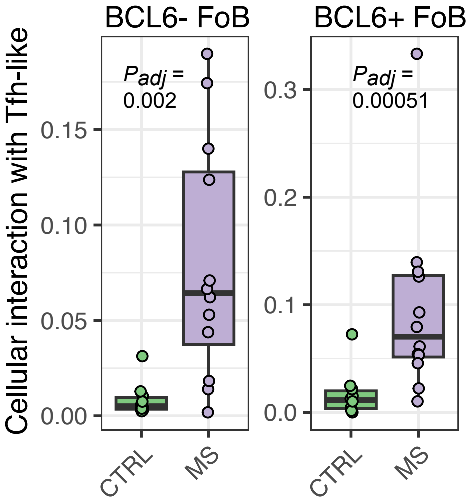

Supplementary Figure 4: Spatial interaction between Tfh-like cells and FoB cells using box plots. P-values are BH-corrected after an unpaired Wilcoxon test.

Supplementary Figure 5

A.

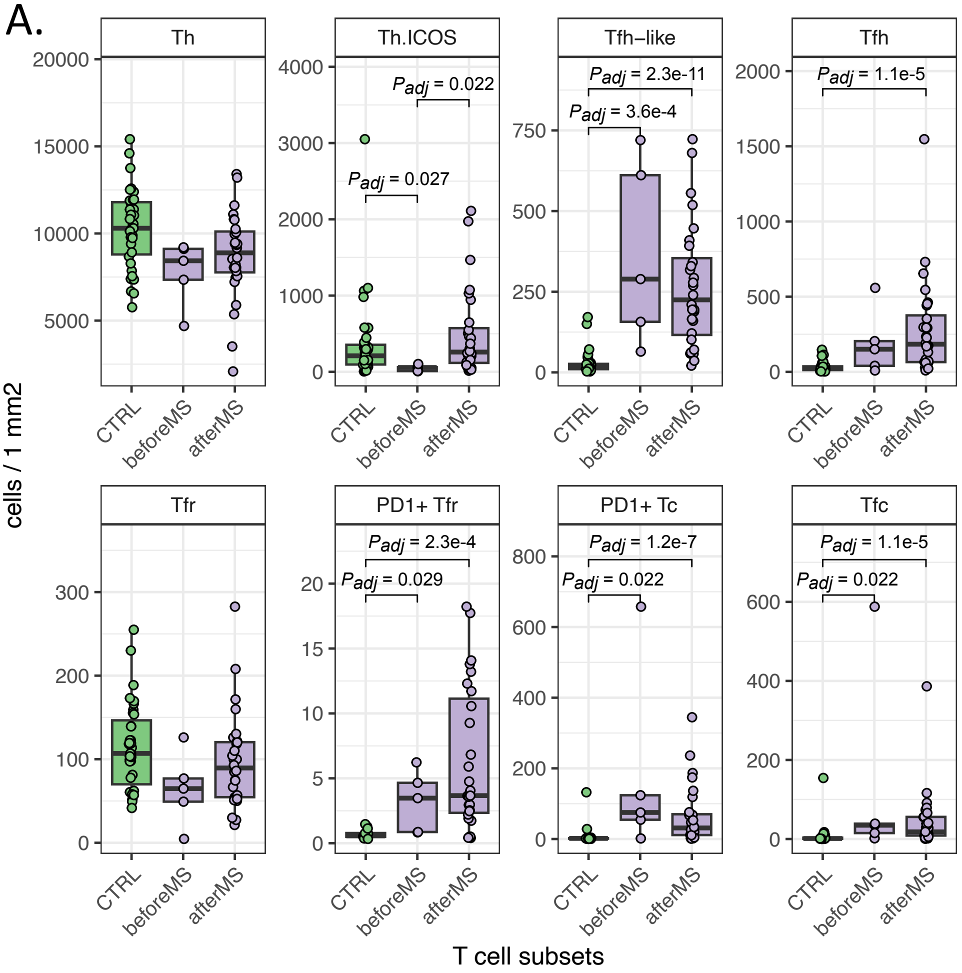

B.

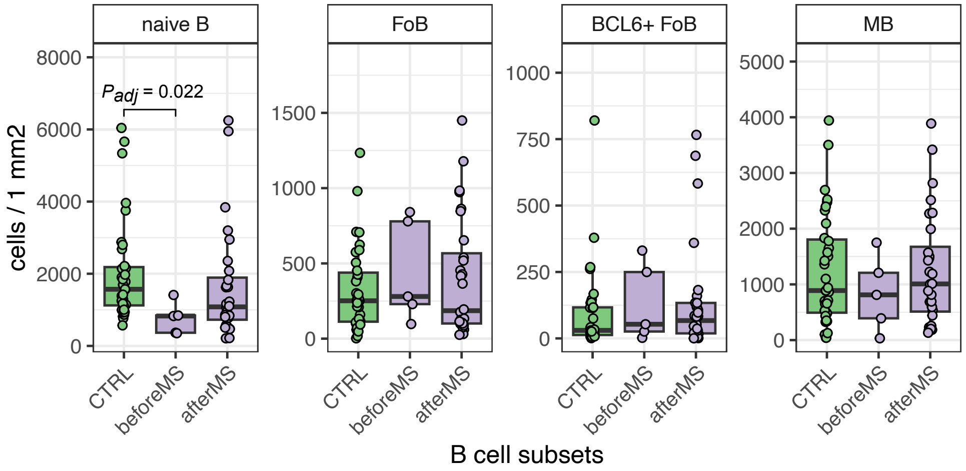

C.

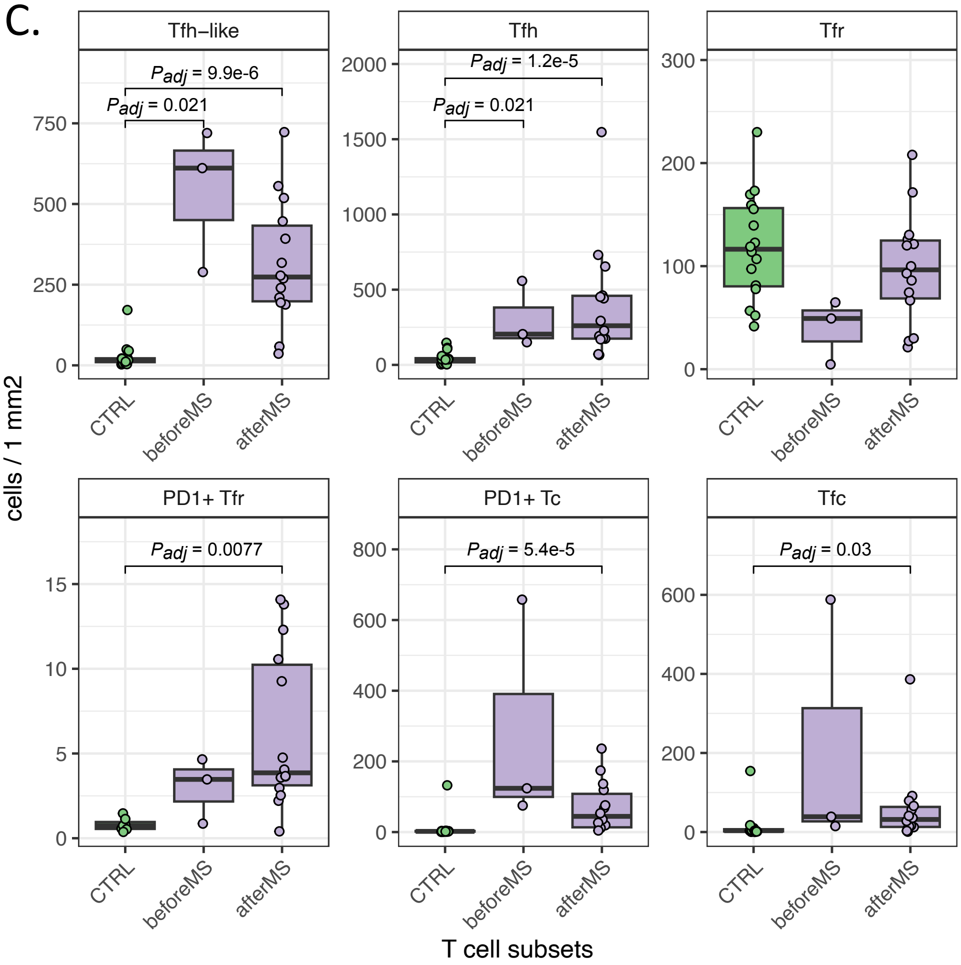

D.

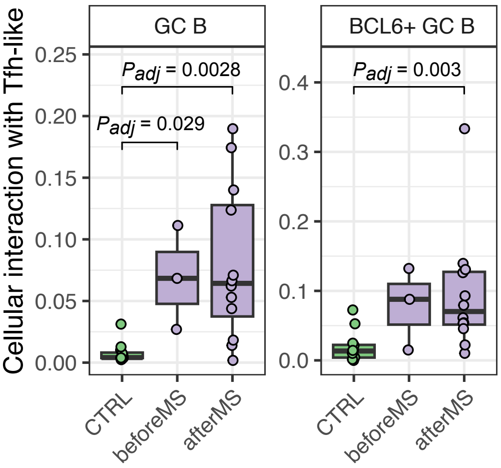

Supplementary Figure 5: Tfh cells are increased in prediagnostic MS LNs. (A-C) Box plots between controls, prediagnostic MS samples and pwMS showing number of (A) T cell subsets, (B) B cell subsets, and (C) T cell subsets in samples with active GCs. **(D)** Spatial interactions between Tfh-like cells and BCL6+/- GC B cells in follicles with active GCs using vbox plots. For all, the BH corrected unpaired Wilcoxon test was used and p-values < 0.05 are shown.

Supplementary Figure 6

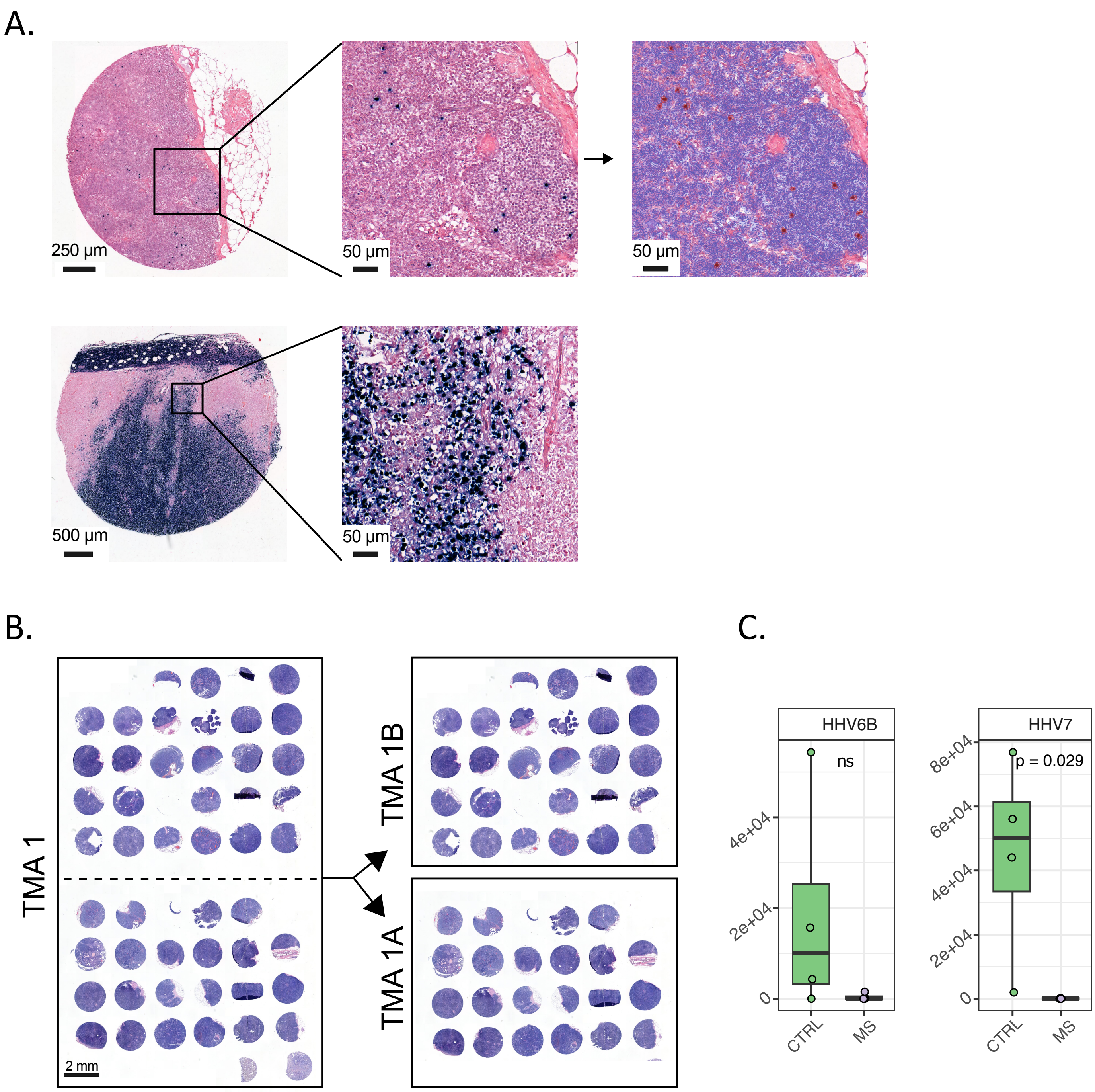

Supplementary Figure 6: Viral assays. (A) Images of EBER-ISH-stained TMA punch from pwMS (top) and positive control (bottom). The top right image highlights EBER-ISH positive cells after analysis with the "Positive cell detection" -tool using QuPath. **(B)** An illustration demonstrates how each TMA was divided into two. The 2 MS and 2 control TMAs resulted with 4 MS and 4 control samples, from which DNA was extracted and analysed using multiplex PCR (HERQ-9). **(C)** Box plots of HHV6B and HHV7 copy numbers per million cells. A Wilcoxon test was used to calculate *P*-values.
